# Sensitive and Quantitative Three-Color Protein Imaging in Fission Yeast Using Spectrally Diverse, Recoded Fluorescent Proteins with Experimentally-Characterized *In Vivo* Maturation Kinetics

**DOI:** 10.1371/journal.pone.0159292

**Published:** 2016-08-01

**Authors:** Bassem Al-Sady, Rachel A. Greenstein, Hana J. El-Samad, Sigurd Braun, Hiten D. Madhani

**Affiliations:** 1 Department of Microbiology and Immunology, the GW Hooper Foundation, University of California San Francisco, San Francisco, California 94143, United States of America; 2 Department of Biochemistry and Biophysics, University of California San Francisco, San Francisco, California 94143, United States of America; 3 TETRAD graduate program, University of California San Francisco, San Francisco, California 94143, United States of America; 4 Department of Physiological Chemistry, Biomedical Center, Ludwigs-Maximilians-University of Munich, 82152 Martinsried, Germany; University of Cambridge, UNITED KINGDOM

## Abstract

*Schizosaccharomyces pombe* is an outstanding model organism for cell biological investigations, yet the range of useful and well-characterized fluorescent proteins (XFPs) is limited. We generated and characterized three recoded fluorescent proteins for 3-color analysis in *S*.*pombe*, Super-folder GFP, monomeric Kusabira Orange 2 and E2Crimson. Upon optimization and expression in *S*. *pombe*, the three proteins enabled sensitive simultaneous 3-color detection capability. Furthermore, we describe a strategy that combines a pulse-chase approach and mathematical modeling to quantify the maturation kinetics of these proteins *in vivo*. We observed maturation kinetics in *S*. *pombe* that are expected from those described for these proteins *in vitro* and/or in other cell types, but also unpredicted behaviors. Our studies provide a kinetically-characterized, integrated three-color XFP toolbox for *S*. *pombe*.

## Introduction

Visualizing the relationships between cellular structures in vivo requires the ability to tag and image multiple proteins at once. This is generally accomplished using engineered fluorescent proteins (XFPs)[1]. The fission yeast *S*. *pombe* is a premier system for investigations of cell biology. Surprisingly, only a few XFPs have been fully validated for dynamic analysis in *S*.*pombe*. In addition, the choice for characterized and optimized fluorescent proteins that are suitable for multicolor analysis is limited compared to the arsenal available for *Saccharomyces cerevisiae* [2]. Most studies in *S*.*pombe* focus on protein fusions to either GFP or YFP. There are few reports of red/orange (mostly mCherry [3–8]) and very few reports of use of blue [9] or far-red fluorescent proteins [3]. The commonly used fluors allow little room for the spectral separation required for robust and specific 3-color detection. The most common combination for imaging in *S*.*pombe*, GFP and mCherry, would require a blue protein as a third color, which commonly displays limited brightness. In addition to blue, green and red proteins, a typical combination for spectrally separable 3-color detection is green, orange and far-red. To date, there is only one report to our knowledge of the use of orange and far-red proteins in *S*.*pombe*, primarily as fusion proteins for cytoskeletal analysis[3].

To address this unmet need, we sought to develop a set of XFPs in *S*.*pombe* that combines the following characteristics: 1) good spectral separation to allow flow cytometric and microscopic analysis of three colors, 2) low limits of detection even in three color tracking settings that may not be fully optimized for any one color. Given the slow maturation kinetics and low photo stability of some of the initial XFP isolates [10, 11], much of the protein engineering effort has gone to optimizing those parameters [12–15]. Maturation kinetics are of particular importance in the context of imaging dynamic processes. Long maturation times make challenging the investigation of the dynamics of a biological process that occur on relatively short timescales. Maturation times are rarely measured for the system of study [16], but rather typically inferred from *E*.*coli* expression or *in vitro* refolding measurements [13, 17–19]. However, it is becoming increasingly evident that the biological environment influences fluorophore maturation, with differences documented even between strains of the same organism [20]. This may not be surprising given the known dependence of maturation on pH and oxygen environment, temperature [21–24] and probably others.

While determining the maturing rate *in vivo* is important, it is not trivial. Most previous approaches to extract maturation times rely on measuring *in vitro* or cell-free refolding rates [13, 15, 18], which may not apply *in vivo*, or approaches which require administration of an efficient translation block under constitutive expression. The latter method, while compatible with *in vivo* settings, requires the ability to measure small increases in fluorescence [20, 25]. *E*.*coli* is the primary system where transcriptional initiation is combined with a chloramphenicol translation block to measure maturation time [12], relying on IPTG as the inducer. While practical, this system is not available for eukaryotes. Further, since such studies only measure fluorescence, they do not take into account the time lag between induction and translation, yielding a compound rate. An alternative method to estimate maturation time, given the requirement for molecular oxygen for the fluor-forming chemistry, involves re-oxygenation after hypoxia [26, 27]. However, this method only addresses the very last step of maturation, fluor cyclization, and may represent an underestimate of maturation kinetics.

**Fig 1:**
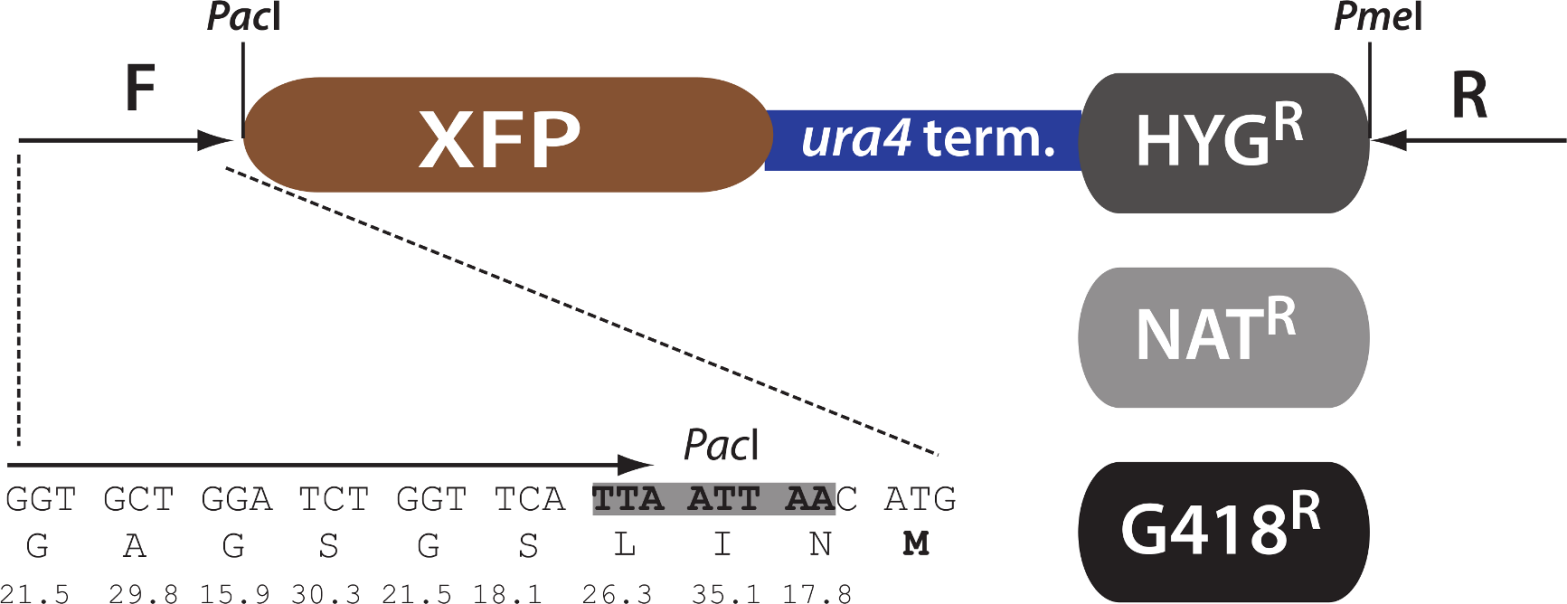
C-terminal tagging vectors for *Schizosaccharomyces pombe* optimized XFPs. SF-GFP, mKO2 and E2C open reading frames were optimized for expression in *S*.*pombe*. The ORFs are preceded by a Gly rich linker (*S*.*pombe* codon frequency of linker amino acids below) and a PacI restriction site. The XFP is followed by the *ura4* terminator and a hyg, nat or G418 MX resistance cassette, followed by a PmeI restriction site. For easy of cloning, a common forward (F) and reverse (R) can be used to amplify any of the 9 XFP:ura4t:MX constructs.

We report here a recoded, optimized set of XFPs for *S*. *pombe*, that offers sensitive detection, simultaneous three-color detection, and for which we have determined the *in vivo* folding kinetics using a novel approach. This new XFP toolbox will enable quantitative and dynamic studies of *S*. *pombe* biology. We standardized our optimized XFP toolbox as C-terminal tagging vectors (Fig 1) and deposited relevant plasmids to Addgene for easy community access.

## Results

### Optimization of SF-GFP mKO2 and E2C for use in *S*.*pombe*

**Fig 2:**
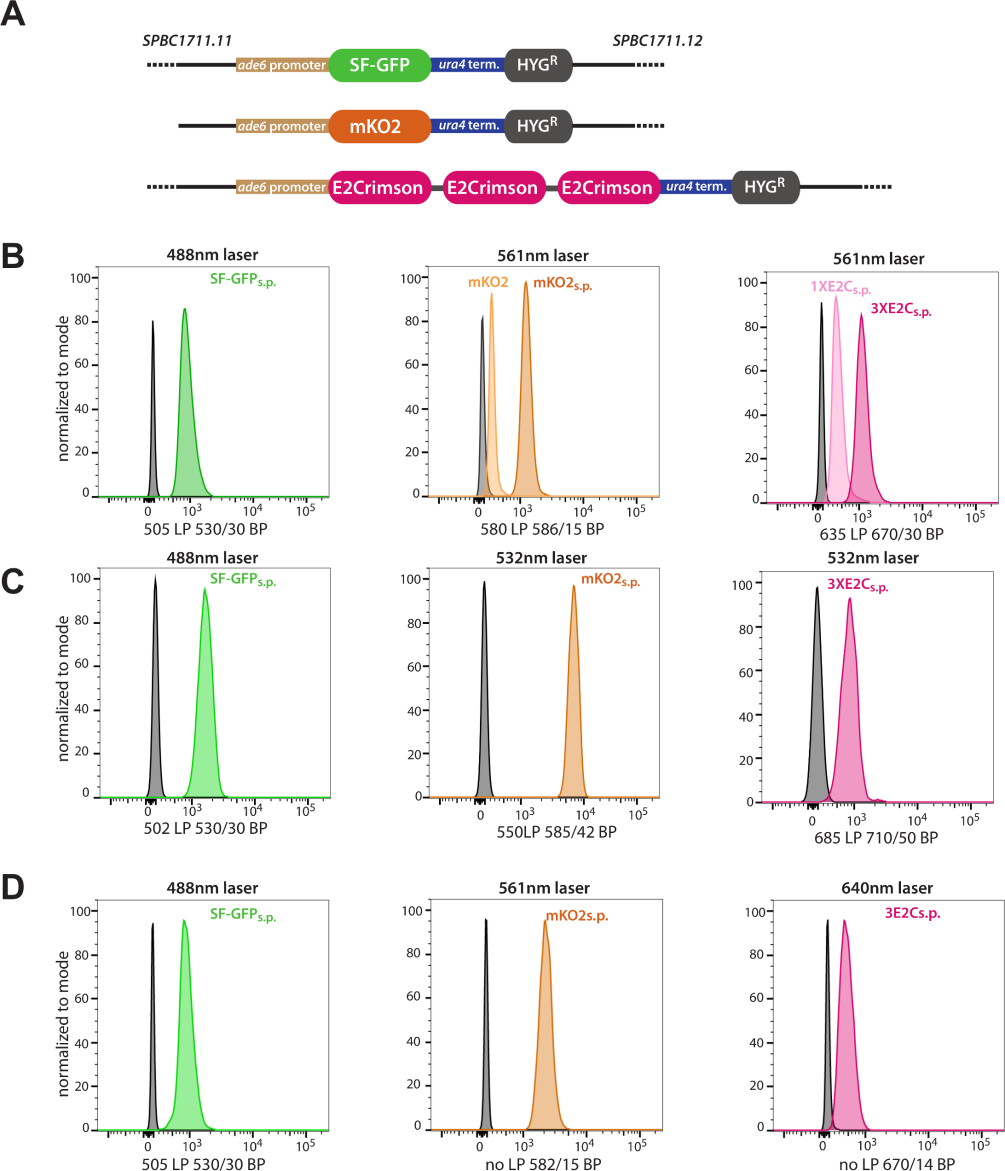
Detection of *ade6* promoter-driven SF-GFP_s.p._, mKO2_s.p._ and 3XE2C_s.p._ by flow cytometry. **A.** Constructs driving on of the three XFPs from the weak *ade6* promoter, terminated by the *ura4* terminator and marked by a hyg resistance cassette were inserted on chromosome II between *SPBC1711*.*11* and *SPBC1711*.*12*. A trimerized version of E2C was used. **B.** The three strains were examined using a 488nm to excite SF-GFP_s.p._ and 561nm laser to excite mKO2_s.p._ and 3XE2C_s.p._ The effect of coding message optimization is shown for mKO2, where the non-optimized version (mKO2) is only 25% as bright as the optimized version (mKO2_s.p._). A 2.7- fold increase in signal by trimerizing E2C (3XE2C_s.p._) versus the monomeric version ((1XE2C_s.p._). **C.** Excitation with 488nm for SF-GFP_s.p._ and 532nm for mKO2_s.p._ and 3XE2C_s.p._. 532 excitation results in a ~10 fold increase of mKO_s.p._ signal versus excitation at 561nm. **D.** Excitation with 488nm for SF-GFP_s.p.,_ 561nm for mKO2_s.p._ and 640nm for 3XE2C_s.p._. The analysis was performed using two flow cytometers, the emission filter sets are denoted below the histograms.

In order to assess the usefulness in *S*.*pombe* of the XFPs we were testing, we expressed them under control of the *bona fide* weak promoter of the *S*.*pombe ade6* gene and assessed their activity initially by flow cytometry [28]. Using a weak promoter as a base to optimize the system provides a realistic benchmark for wider applicability. All XFPs tested were inserted (*ade6p*:XFP:*ura4t*) as a part of a hygromycin resistance cassette in chromosome II between *SPBC1711*.*1* and *SPBC1711*.*12* (Fig 2A, see Table 1 for all strain genotypes). We initially tested the expression of a range of XFPs, with their messages not optimized for codon usage, with focus on proteins reported to be rapidly maturing, and that in combination have significant spectral separation: mTagBFP2[29], turbo GFP[30], SF-GFP[13], YFP-Venus[16], mKO2[31], TagRFP-T[14], mOrange2 [14], mcherry, mKate2[32] and E2Crimson[33]. Of those, mTagBFP2, turbo GFP and, surprisingly, YFP-Venus gave no detectable signal by flow cytometry (data not shown). mKate2 and mOrange2 gave only weak signal, while SF-GFP, TagRFP-T, mKO2, E2Crimson and mCherry have low to intermediate signals (Fig 2B). SF-GFP, mKO2 or TagRFP-T and E2Crinsom form a compatible 3-color detection set. Since in our hands mKO2 was significantly brighter than TagRFP-T, we moved forward with optimizing the coding messages of SF-GFP, mKO2 and E2C XFPs for the codon usage and GC content of *S*.*pombe* (proprietary GeneOptimizer algorithm, Life Technologies). We denote these versions with the subscript s.p. The alignment between pre-optimized XFPs and s.p. XFPs can be found in S File 1. Message optimization resulted in a substantial increase in XFP fluorescence as measured by flow cytometry signals (see for mKO2, Fig 2B). Using a 488nm laser to excite SF-GFP, and a 561nm laser to excite both mKO2 and E2C, we obtained about 10-15-fold signal over background (untransformed cells) for SF-GFP_s.p._ and mKO2_s.p._, but only around 4-fold for E2C_s.p._. To increase E2C signals to a similar level as the other two XFPs under these detection conditions, we fused three E2C monomers separated by Glycine-Serine linkers between the three units. This triple protein (3xE2C_s.p._) yielded roughly 2.7x the signal of the monomer, resulting in around 12-fold signal to noise.

**Table 1:** *Schizosaccharomyces pombe* strain genotypes used in this study.

Strain	Genotype
BAS99	*h+ ura4-D18 ade6-M210 leu1-32 smt0*
BAS42	*h+ SPBC1711*.*11*:: *ade6p*:*mKO2*:*ura4t*:*hygMX ura4-D18 ade6-M210 leu1-32 smt0*
BAS52	*h+ SPBC1711*.*11*:: *ade6p*:*mKO2*_*s*.*p*._:*ura4t*:*hygMX ura4-D18 ade6-M210 leu1-32 smt0*
BAS55	*h+ SPBC1711*.*11*:: *ade6p*:*SF-GFP*_*s*.*p*._:*ura4t*:*hygMX ura4-D18 ade6-M210 leu1-32 smt0*
BAS65	*h+ SPBC1711*.*11*:: *ade6p*:*E2C*_*s*.*p*._:*ura4t*:*hygMX ura4-D18 ade6-M210 leu1-32 smt0*
BAS75	*h+ SPBC1711*.*11*:: *ade6p*:*3xE2C*_*s*.*p*._:*ura4t*:*hygMX ura4-D18 ade6-M210 leu1-32 smt0*
BAS138	*h- urg1Δ*::*kanMX*
BAS132	*h- urg1Δ*::*urg1p*:*SF-GFP*_*s*.*p*_:*5xFLAG*:*urg1 3’UTR*:*natMX*
BAS139	*h- urg1Δ*::*urg1p*:*mKO2*_*s*.*p*_:*5xFLAG*:*urg1 3’UTR*:*natMX*
BAS142	*h- urg1Δ*::*urg1p*:*E2C*_*s*.*p*_:*5xFLAG*:*urg1 3’UTR*:*natMX*
BAS208	*h- swi6*::*swi6*:*SF-GFP*_*s*.*p*._:*ura4t*:*G418MX ura4-D18 ade6-M210 leu1-32 smt0*
BAS209	*h- SPBC1711*.*11*:: *act1p*: *E2C*_*s*.*p*._:*ura4t*:*hygMX ura4-D18 ade6-M210 leu1-32 smt0*
BAS210	*h+ sad1*::*sad1*:*mKO2*_*s*.*p*._:*ura4t*:*NatMX ura4-D18 ade6-M210 leu1-32 smt0*
BAS211	*h- swi6*::*swi6*:*E2C*_*s*.*p*._:*ura4t*:*HygMX ura4-D18 ade6-M210 leu1-32 smt0*
BAS224	*swi6*::*swi6*:*E2C*_*s*.*p*._:*ura4t*:*HygMX; sad1*::*sad1*:*mKO2*_*s*.*p*._:*ura4t*:*NatMX ura4-D18 ade6-M210 leu1-32 smt0*

To make the optimized XFPs available to the community in a multiuse format, we produced C-terminal tagging vectors for the monomeric versions of E2C_s.p._, SF-GFP_s.p._ and mKO2_s.p._. The general format of the vectors is described in Fig 1: The XFP is terminated by the *ura4* terminator followed by a hygromycin B (hyg), nourseothricin (nat) or G418 (kan) MX drug resistance cassette [6]. The N terminal ATG is preceded by a glycine rich and common linker sequence, allowing for the amplification of each XFP with a common primer. The corresponding reverse primer anneals to all three drug resistance cassettes. The plasmids have been deposited to Addgene (ID numbers 74080–74087).

### Simultaneous detection of SF-GFP_s.p._ mKO2_s.p._ and E2C_s.p._ by flow cytometry

Next, to examine the usefulness of our optimized proteins in three color detection by flow cytometry, we examined the signal to noise in the preferred emission channel (Fig 2) but also the emission bleed from one XFP into the channel of another. We chose three laser excitation scenarios that likely cover most available standard flow cytometer configurations. Briefly, in all experiments, SF-GFP is excited by a 488nm laser, available on most stand cytometers, while mKO2 and E2C proteins are excited by either 532nm, 561nm or 640nm lasers. Under a configuration where SF-GFP_s.p._ is excited by a 488nm laser, and mKO2_s.p._ and 3xE2C_s.p._ are exited with a 561nm laser (488/561/561), we obtain about 10-15x signal for the three XFPs relative to untransformed control cells (Fig 2B). If instead we excited mKO2_s.p._ and 3xE2C_s.p._with a 532nm laser (488/532/532), we can obtain almost 100x signal over noise for mKO2_s.p._, at the cost of decrease in signal for 3xE2C_s.p._(Fig 2C). This configuration is useful if one of the proteins under study is expressed/produced at a lower level than the others. For such a low-abundance protein, mKO2_s.p._ is ideally suited as it is significantly brighter at its optimal excitation than SF-GFP_s.p._ or 3xE2C_s.p._ To assess bleed and compensation, we stayed with the 488/561/561 excitation regime that produces even signal for all three proteins. Neither SF-GFP_s.p._ nor 3xE2C_s.p._ exhibits any significant bleed into the channels for the other XFPs (S1 Fig). On the other hand, mKO2_s.p._ has very minor bleed into the GFP channel, and significant bleed into the E2C channel (S1 Fig, left). This emission light bleed can be efficiently compensated by common cytometry software packages such as the DIVA software on BD flow cytometers (S1 Fig, right), allowing specific detection of all three XFPs simultaneously. It is also possible to detect 3xE2C_s.p._ using a 640nm laser (488/532/640 or 488/561/640). In the 488/561/640 excitation regime (Fig 2D), there is no detectable bleed from mKO2 into the E2C 640nm excited, 670/14 channel. The only compensation required in these conditions therefore is for the slight bleed from mKO2_s.p._ into the GFP channel (S2 Fig). We found that under the conditions of this experiment, the 3xE2C_s.p._ signal was reduced about 40% compared to excitation at 561nm. The 488/561/640nm excitation combination is the most specific, with little compromise in signal-to-noise versus 488/561/561nm. However, if this combination is not available, we have shown here that the 3x-color system can be specifically detected using alternative (488/532/532; 488/561/561) combinations.

### Determining maturation times of XFPs in live *S*.*pombe* cells

SF-GFP[13], E2C[33] and mKO2[26, 31] have been described as fast maturing proteins, yet this was established either *in vitro* or in heterologous systems. It has remained unclear whether maturation kinetics derived in one system or even strain[20] will hold for the intended system of study. To address this uncertainty, we devised an experimental method to determine the *in vivo* maturation time of XFPs in *S*.*pombe*. Conceptually, our system requires only two key factors: The ability to induce expression of an XFP cassette, preferably producing an unstable message, and second, the ability to follow the protein and fluorescence accumulation of the XFP. In that sense, our method should have wide applicability to other biological systems.

**Fig 3:**
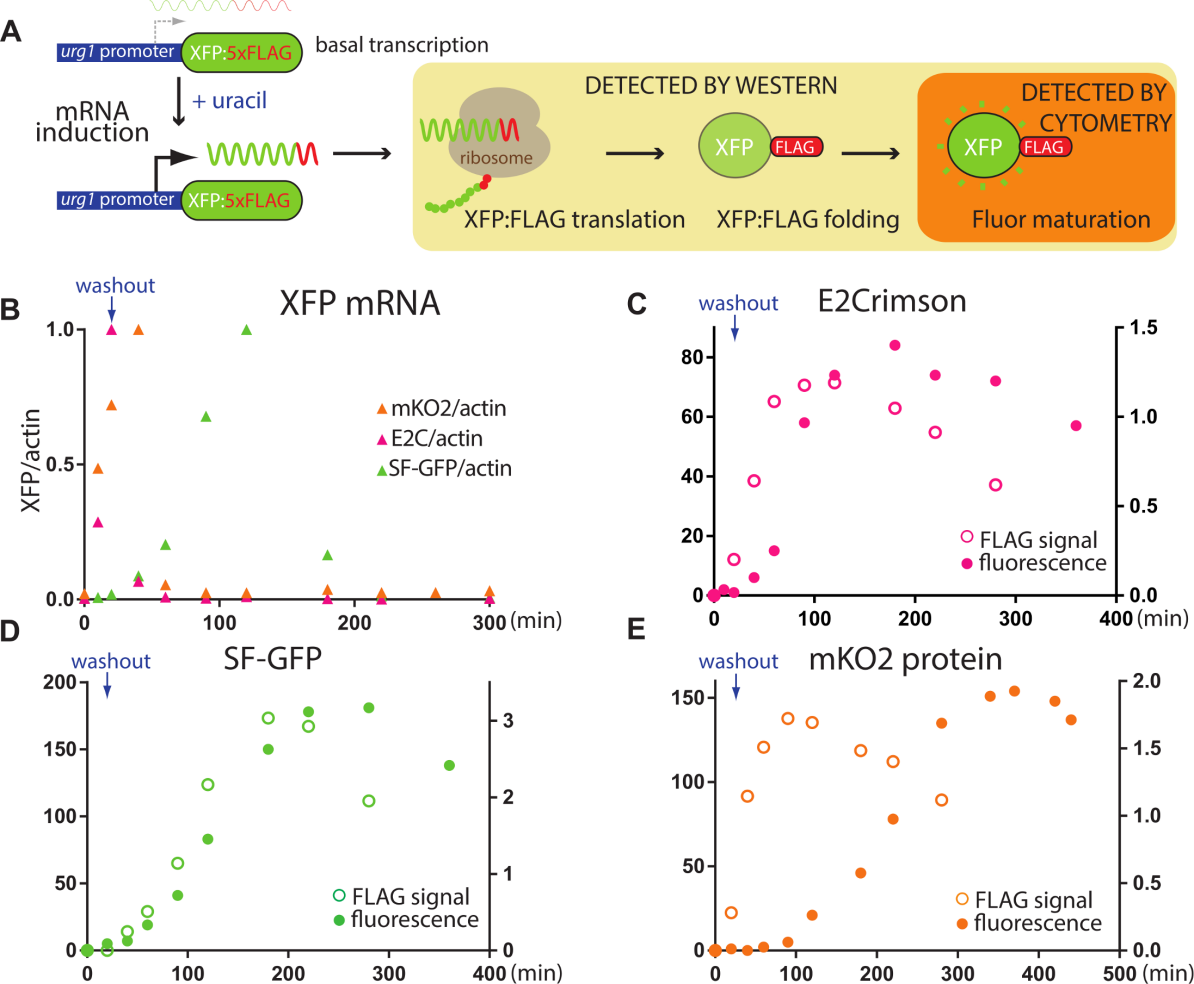
**Determination of maturation kinetics *in vivo* of SF-GFP_s.p._, mKO2_s.p._ and E2C_s.p._ A. Overview of approach.** XFPs were placed downstream of the *urg1* promoter and tagged with a 5XFLAG tag. Transcription was induced by uracil for 20min. Anti-FLAG westerns can detect all stages following translation including folding and maturation (light orange box), while follow cytometry measurements only detect the fully matured protein. **B. The dynamics of XFP:5xFLAG transcripts following the removal of uracil.** E2C_s.p._ / actin, SF-GFP_s.p._ / actin and mKO2_s.p._ / actin mRNA rations are plotted as function of time and the peak set to 1 for each of the fluors. Uracil was administered from 0-20min. E2C_s.p._ transcript peaks at 20min, mKO2_s.p._ peaks at 40min, while SF-GFP_s.p._ peaks at 120min. **C.-E. Time courses of total protein and fluorescence.** The GAPDH normalized FLAG western blot signal (ng protein) is plotted on the right axis, total fluorescence as measured by flow cytometry on the left axis.

We use the well-described uracil-responsive gene *urg1* [34, 35] in *S*.*pombe* to drive production of our XFPs in response to uracil administration. Conceptually, this system is very similar to induction of gene expression in response to auxotrophic media conditions in *S*.*cerevisiae* [36]. One striking and useful feature of the *urg1* system is the rapid decay of the message following withdrawal of uracil [35], allowing for production of a XFP pulse that can be followed. Genes encoding the three fluors C-terminally tagged with a 5x FLAG tag were placed downstream of the *urg1* promoter, replacing the endogenous *Urg1* ORF (Fig 3A). Cells were grown to early log–phase, and XFP production was induced with uracil for 20min followed by a uracil-free chase. To assess uracil induction and decay kinetics of our *urg1p*:XFP:5xFLAG messages, we tracked them by RT-qPCR over time (Fig 3B). We find that all messages are induced strongly by uracil and exhibit a sharp drop following their induction peak. However, while mKO2 and E2C mRNAs are induced rapidly, with messages peaking at 40min and 20min, respectively, induction of SF-GFP mRNA is delayed, and peaks at around 120min. This delay may be due to an altered transcriptional response or chromatin state at the *urg1* promoter in the *urg1p*:SF-GFP:5xFLAG strain. While SF-GFP induction exhibits a delayed response to uracil, mKO2 and E2C mRNA exhibit a very rapid response and decay as published for the endogenous *urg1* gene [35]. Since all three messages respond to uracil with a pulse of mRNA production, it renders them equally useful for our maturation experiments.

To obtain an estimate for XFP folding and maturation following translation, we reasoned that western blot detection of the XFP:FLAG protein would capture all stages following translation, while fluorescence detection of the XFP would capture only the fully matured state (see scheme Fig 3A). We measured protein production using LiCor western blots, detecting FLAG and cellular GAPDH control signals simultaneously. The same samples were measured at indicated time points for fluorescence using the flow cytometry setup as in Fig 2B (Fig 3C–3E). In order to determine the maturation time of our three XFPs, we took the following approaches:

1. To estimate the delay between protein appearance and fluorescence, the folding maturation half-time (t_1/2 fold/mature_), we subtract the half-time of protein production as estimated by western blots (t_1/2 protein_), from the half-time of appearance of maximal fluorescence ((t_1/2 fluorescence_), i.e. t_1/2 fold/mature_ = t_1/2 fluorescence_—t_1/2 protein_. We estimate the half time from the point of apparent maximal FLAG signal of fluorescence signal (see Table 2). Interestingly, we find that t_1/2 protein_ is not identical between the three XFPs: It is similar between *urg1p*:mKO_s.p._ and *urg1p*:E2C_s.p._, but apparently slower for *urg1p*:SF-GFP_s.p._ (Fig 3D, and see below). As noted above, this difference is due to a longer lag in uracil response of the SF-GFP_s.p_ mRNA in the *urg1p*:SF-GFP_s.p._ strain. However, this delay in SF-GFP_s.p._ protein appearance caused by delayed induction of the message, relative to the other two fluors, underscoring the importance of measuring protein translation/folding rates in response to an inducer. If we now subtract this t_1/2 protein_ from t_1/2 fluorescence_, we obtain a t_1/2 fold/mature_ of 25min for SF-GFP and 40min for E2C. However, the t_1/2 fold/mature_ for mKO2_s.p._ is significantly longer, at ~135min. This does not match with its previous characterization as a fast folding protein [26, 31] and may indicate that the intracellular environment in *S*.*pombe* imposes slower maturation kinetics for mKO2.

**Table 2:** Halftimes of maximal protein or fluorescence accumulation.

XFP	~ t_1/2_ max protein	~ t_1/2_ max. fluorescence	t_1/2_ fold/mature
SF-GFP	90min	115min	25min
E2C	40min	80min	40min
mKO2	40min	175min	135min

2. Next, we aimed to extract the *in vivo* intrinsic rate constants of both protein production following induction and folding/maturation from our data (see Table 3). In our initial model, we used two protein states:

**Table 3:** Fitting-derived parameters.

XFP	*u*	*α*_*M*_ (min^-1^)	*k*_*P*_ (min^-1^)	*α*_*P*_ (min^-1^)	*k*_*FM*_ (min^-1^)	*β*_*FM*_ (min^-1^)	*θ*	*k*_*o*_ (min^-1^)	*k*_*c*_ (min^-1^)
E2C	**3.75**	**0.008**	**0.0004**	**0.0097**	**0.008**	**0.002**	**115**	**N/A**	**N/A**
SF-GFP	**0.26**	**0.009**	**0.0002**	**0.011**	**0.019**	**0.005**	**91.1**	**12.1**	**0.009**
mKO2	**3.9**	**0.0035**	**0.0007**	**0.018**	**0.0047 (*k***_***F***_**) 0.0052 (*k***_***M***_**)**	**0.0018**	**373.8**	**N/A**	**N/A**

RNA➔ translated protein (*P*_*T*_)➔ folded/matured protein (*P*_*FM*_).

**Fig 4:**
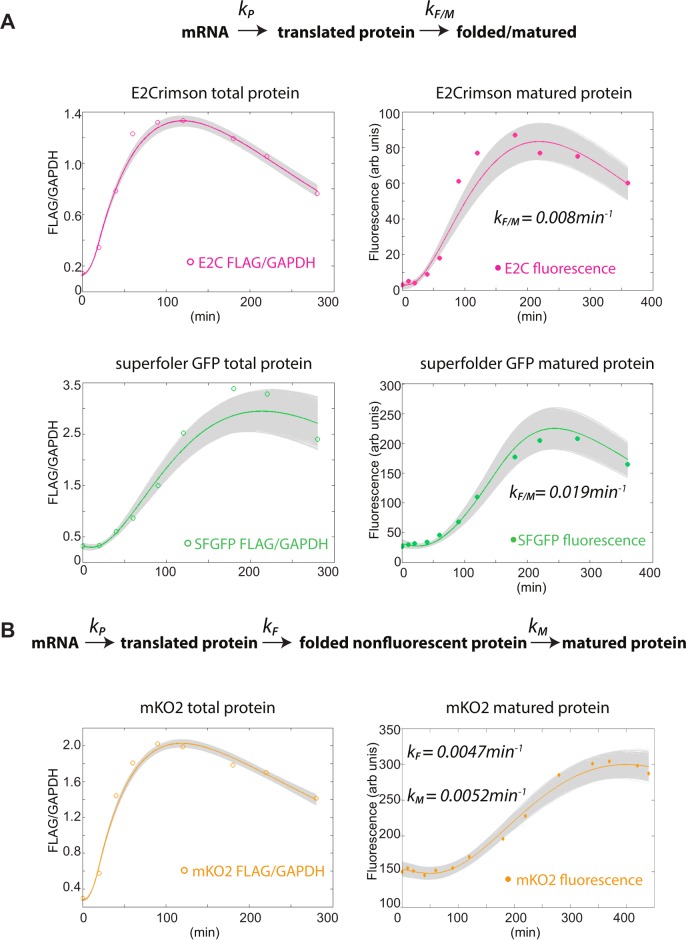
Mathematical fitting of total protein (FLAG/GAPDH) and mature protein (fluorescence) data. **A.** E2C and SF-GFP fits assume a two protein state model (translated and folded/matured). The SF-GFP protein fit assumed an equilibrium between a closed (non-uracil responsive) and open (uracil responsive) chromatin state at the *urg1* locus. **B.** mKO2 fits assume a three protein state model (translated, folded and matured). The solid lines represents the mean of all possible fits to the model, the gray lines represent all the fits with parameter combinations yielding less than 5% mean fitting error.

We extracted the rate constants of XFP production and folding/maturation by applying a simple set of differential equations with one rate constant for production, *k*_*P*_, and one for folding/maturation *k*_*FM*_ (Fig 4A). For E2C_s.p._ and mKO2 _s.p._, *k*_*P*_ could be directly extracted from the data by fitting equations *(1) (2)* described in the fitting methods section. For both these fluors, the fit of the protein production equations to FLAG western data was excellent, indicating that the transcription and translation of the fluorescent proteins can be explained by the most parsimonious simple linear model with no rate limiting steps (Fig 4A top and 4C). By contrast, we could not fit the FLAG western data for SF-GFP_s.p._ with these simple equations. As noted above, the *urg1p*:SF-GFP_s.p._ strain responded slower to uracil administration, evident in the delay in message (and protein) appearance relative to E2C and mKO2 (Figs 3 and 4). Together with our observations that this reporter gene becomes repressed over long generational times (data not shown), we believe this delay to be due to an equilibrium between an accessible (open) and inaccessible (closed) chromatin state. We therefore introduced an additional equation that modeled explicitly such an equilibrium, resulting in a good fit of the SF-GFP_s.p._ protein data (Fig 4A bottom). We obtained a translation rate *k*_*P*_ for SF-GFP_s.p._, E2C_s.p._ and mKO2_s.p._ of 0.0002min^-1^, 0.0004min^-1^ and 0.0007min^-1^, respectively.

To fit the fluorescence data, we opted to feed the measured FLAG western data directly into the fluor folding/maturation equation (Fig 4A). Decoupling the fit of the total protein from that of the fluorescent pool makes the extraction of the folding/maturation parameters independent of the quality of fit of the protein. The simple two-state model generated excellent fits for SF-GFP_s.p._ and E2C_s.p._ (methods), generating folding/maturation rate *k*_*FM*_ of and 0.019min^-1^ and 0.008 min^-1^, respectively, indicating a faster folding/maturation rate for SF-GFP_s.p._. This is consistent with the shorter t_1/2 fold/mature_ for SF-GFP_s.p._. Importantly, to test the robustness of the extracted parameters, we used a procedure in which we perturbed the fit parameters and simulated the equations with these parameters, keeping only fits that resulted in less than 5% mean fitting error from the data. S3A and S3B Fig shows the distributions of the resulting parameters, illustrating that different parameter combinations are indeed capable of reproducing the data at this tolerance, but that these values were closely centered around the mean fit values which we report.

Using the same procedure, we were not able to find any parameter set that can fit the mKO2 fluorescence data for the two protein state model in which a single step described folding and maturation. On inspection of the raw data, we noticed a long lag between maximal accumulation of translated protein and the appearance of any fluorescence in two replicates of the experiment (Fig 4D and S4A Fig). We reasoned that the observed long lag with zero accumulation of fluorescence is not consistent with a model where the maturation rate is the sole limiting rate following translation, but rather indicates another, non-fluorescent mKO2_s.p._ intermediate formed at a slow production rate. To account for this lag and a possible non-fluorescent intermediate, we proceeded to fit the data to the two protein state model but including a 100 min delay following the translation step. This approach resulted in an acceptable fit (S4B Fig), with a fitted *k*_FM*_ of 0.00015min^-1^. Since we can only obtain a *k*_*FM*_ with the introduction of a long delay function, we hypothesized that formation of this the non-fluorescent intermediate takes place over significant timescales and thus expanded the model to a three protein state model:

RNA➔ translated protein (*P*_*T*_)➔ folded protein (*P*_*F*_) ➔ mature protein (*P*_*M*_) (Equation in Fig 4B). Using this model, we could fit the data, extracting a separate folding constant *k*_*F*_ and maturation constant *k*_*M*_ values of 0.0047min^-1^ and 0.0052min^-1^, respectively (Fig 4C). These values were consistent in a full independent repeat of the entire experiment (S4A Fig). Even though the *k*_*M*_ is not drastically different from above *k*_*FM*_ values for SF-GFP_s.p._ and E2C_s.p._, the preceding rate *k*_*F*_ of formation of the non-fluorescent intermediate is on the same timescale as the separate maturation rate, introducing a delay. Together, folding and maturation yield a much higher t_1/2 fold/mature_ for mKO_s.p._. It is important to note that the fit with the three state protein model was superior to the one with a simple delay (S4B Fig), highlighting that the dynamics of the non-fluorescent intermediate are important to account for. Finally, as in the case for SF-GFP_s.p._ and E2C_s.p._, the parameters that could reproduce the fits at less than 5% mean fitting error clustered very closely around the reported mean (S3C Fig). All the relevant parameters are summarized in Table 3.

In summary, using simple two or three protein state models, we were able to derive combined folding/maturation rates (SF-GFP_s.p._ and E2C_s.p._) or separate folding and maturation rates (mKO2_s.p._) for all three flours in our toolbox, while accounting for the any delays in protein production.

### Usefulness of XFPs for imaging of low-abundance protein in *S*.*pombe* cells

**Fig 5:**
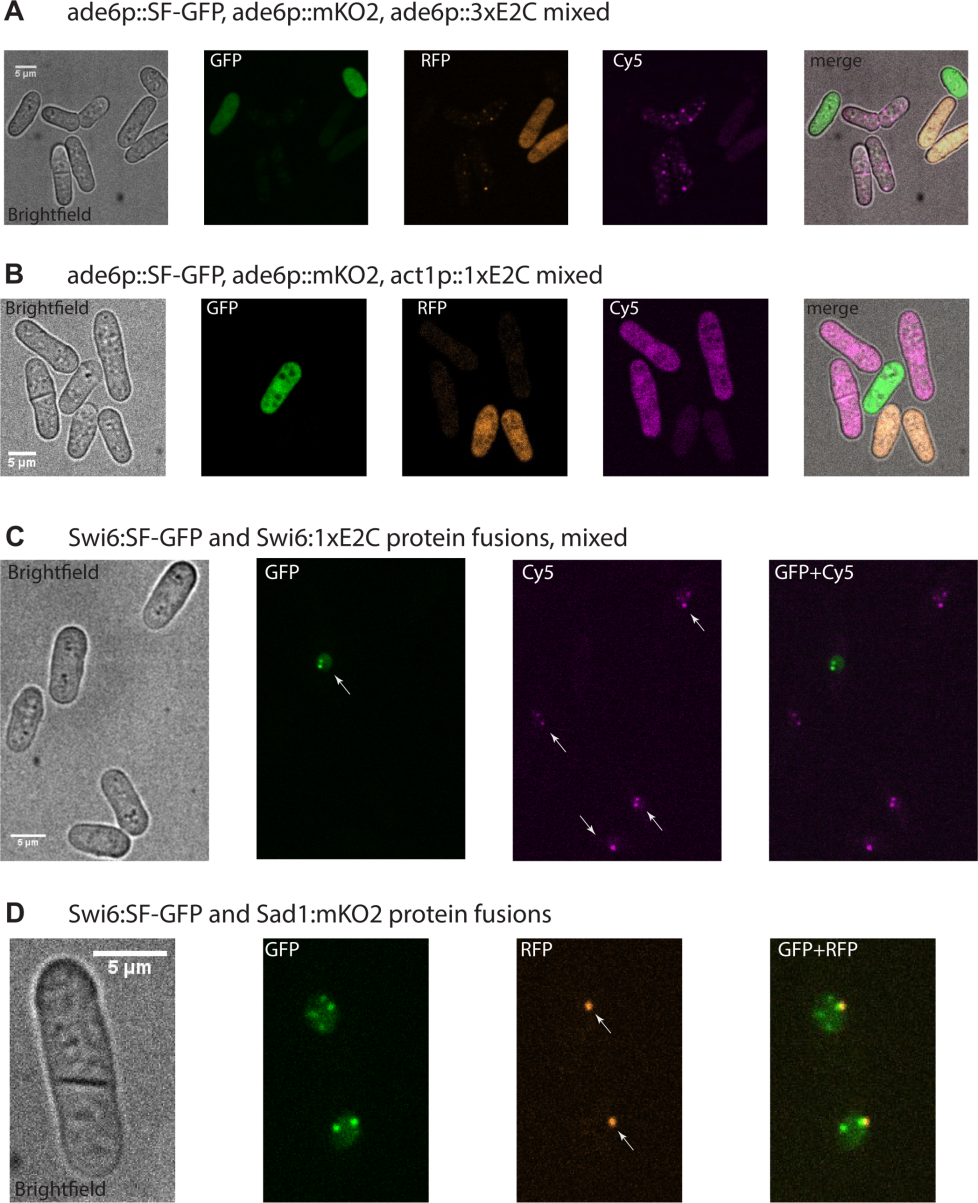
Performance of SF-GFP_s.p._, mKO2_s.p._ and E2C_s.p._ for low abundance protein detection by microscopy. **A.** Untagged *ade6* promoter driven XFPs. SF-GFP_s.p.,_ mKO2_s.p._ and 3XE2C_s.p._ cells were mixed at 1:1:1 and imaged in brightfield and GFP, RFP and Cy5 channels. The merged imaged is shown on the right. Images were taken at 60x magnification. **B.** Untagged *ade6p*:SF-GFP_s.p.,_ *ade6p*:mKO2_s.p._ *act1p*:1xE2C_s.p._ promoter driven XFPs. SF-GFP_s.p.,_ mKO2_s.p._ and 1XE2C_s.p._ cells were mixed at 1:1:1 and imaged cells were visualized in brightfield, the GFP, RFP and Cy5 channels. The merged imaged is shown on the right. Images were taken at 60x magnification. **C.** Swi6 visualized by SF-GFP_s.p._ or 1XE2C_s.p._ Cells containing either Swi6 C-terminally tagged by SF-GFP_s.p._ or 1XE2C_s.p._ were mixed 1:1 and imaged in brightfield and GFP and Cy5 channels. Arrowheads point to cell nuclei. Images were taken at 60x magnification. **D.** Co-localization of Swi6:SF-GFP_s.p._ and Sad1:mKO2 _s.p._ in the same cell. Cells were visualized in brightfield, the GFP and RFP channels. Arrowheads point to Sad1:mKO2 _s.p._-marked spindle pole bodies. Image were taken at 100x magnification.

The *ade6* promoter is fairly weak [28] and thus serves as a useful model of low- to medium low abundant proteins. Using the constructs from Fig 1 (*ade6p*:*XFP*:*ura4t*), we imaged live *S*.*pombe* cells using a spinning disc confocal microscope to address both signal and intracellular distribution of the XFPs alone (Fig 5). The XFPs were excited by a 488nm laser for SF-GFP, 561nm for mKO2 and 640nm for the E2C constructs. While we imaged each strain alone in S5A Fig, in Fig 5A and 5B, we mixed the three strains and co-visualized them under the same microscope settings. Both *ade6p* driven-SF-GFP_s.p._ and mKO2_s.p._ yielded bright signals specifically in their respective emission channels (GFP and RFP, S5A Fig and Fig 5A and 5B), and importantly, show a uniform cellular distribution. *ade6p*:E2C could not be visualized by live-cell microcopy (data not shown), indicating that E2C is less well suited for visualization of low-abundance proteins. In contrast, *ade6p*:3XE2C could be easily visualized in both the Cy5 channel as well as weakly in the RFP channel (S5A Fig and Fig 5A). This is expected given the emission pre-sets in the microscope used and could be eliminated using different filter sets. 3XE2C_s.p._ yielded some diffuse fluorescence background and is excluded from the nucleus. However, it accumulates in several cellular foci. This does not appear to correlate with cellular toxicity as 3XE2C strains grow at the same rate as the untransformed control (data not shown). This aggregation may be a feature of the 3XE2C_s.p._ protein. To test this, we prepared a construct with a 1XE2C_s.p._ driven by the strong *act1* promoter (Fig 5B, Cy5). In this case, we observe strong signal coming from *act1*:1XE2C and importantly, uniform cellular distribution as in the case of SF-GFP_s.p._ and mKO2_s.p._ This observation confirms that the aggregation seen for the *ade6p*:3XE2C is due to the tandem fusion. To determine whether an endogenous protein will display the correct localization pattern when fused to E2C, we C-terminally tagged the heterochromatin protein Swi6 with 1XE2C_s.p._ using the general construct in Fig 1. Employing the same strategy, we fused Swi6 to SF-GFP_s.p._ as a control, since GFP fusions have been previously validated for Swi6 [37]. We observed that both Swi6:SF-GFP_s.p._ and Swi6:1XE2C_s.p._ display the same and correct localization pattern of several nuclear foci and diffuse nuclear staining with no cytosolic fluorescence [38] (Fig 5C). This observation indicates that 1) 1XE2C C-terminal fusions do not interfere with the normal localization of the native protein and 2) moderately expressed proteins such as Swi6 (~10,000 copies per cell) can be visualized with 1XE2C. To explore co-localization of proteins with these tools within the same cell, we fused Sad1, a spindle pole body (SPB) marker present at ~2000–3000 copies per cell, to mKO2_s.p._ and introduced the *sad1*:mKO2_s.p._ construct into the *swi6*:SF-GFP_s.p._ strain. In the RFP channel, we clearly saw a single bright spot marking the SPB (Fig 5D), as expected [39]. In the GFP channel, we saw that Swi6:SF-GFP_s.p._ localizes adjacent to the SPB but also to other nuclear foci that likely represent the heterochromatic telomeric ends; the fusion proteins further exhibits a diffuse nuclear staining (Fig 5D). These data indicate the suitability of our system for co-localization of two or more low- to moderately- expressed proteins in the cell. This is further supported by the little cross bleed we observed even without optimized filter sets for our three fluors.

Our flow cytometry results showed (Fig 1) that consistently, mKO2_s.p._ is the brightest of the three XFPs, and the microscopy analysis indicated that it is readily detected when driven by a low expressed promoter (as is SF-GFP_s.p._). We wondered whether it can be used to visualize a very low abundance protein that could not be visualized when expressed form its own promoter. Dcr1 is a very low expressed protein and could only be visualized when ectopically expressed from a heterologous promoter that is stronger than the endogenous *dcr1* promoter [40]. We constructed an N-terminal mKO2 fusion to Dcr1 (*dcr1p*:mKO2 _s.p._:*Dcr1*:*dcr1t*). We can detect mKO2:Dcr1 over background under this condition, albeit weakly (S5B Fig). This differs from prior attempts, where Dcr1 could only be visualized when expressed from high and intermediate strength versions of a constitutive promoter (*nmt1*) but not its own promoter or weak constitutive versions of *nmt1* [40, 41]. These data indicate that mKO2 _s.p._is particularly well suited as a tag for low expressed proteins in *S*.*pombe*.

## Discussion

In this work we describe an integrated toolbox of XFPs that enables three-color imaging in *S*. *pombe*. We also describe a new method to extract the apparent *in vivo* folding and maturation times for XFPs, which should be widely applicable. We make the following broad observations about the features of the current toolbox: 1. Upon *S*.*pombe* codon optimization, all three fluors are easily detectable by flow cytometry when driven by a weak promoter (*ade6*). 2. The XFPs are spectrally separable, enabling three-color detection in the context of flow cytometry and microscopy. Appropriate filter sets should be easily available to eliminate the remaining bleed from E2C into the RFP channel under the microscope. 3. All three fluors are suitable for localization of endogenous proteins at least when fused C-terminally. 4. SF-GFP and E2C mature relatively quickly *in vivo* in *S*.*pombe*, with maturation halftimes of 25 and 40min, respectively. On the other hand, mKO2 has a longer maturation halftime of around 135min. We believe this is largely due to a long-lived non-fluorescent intermediate state we model as a folded state (see fitting in Fig 4D). 5. SF-GFP and mKO2 are ideal for microscopy imaging for low abundance proteins, as they are easily detected when driven by the *ade6* promoter. E2C is more appropriate for moderate- to high- abundance proteins as we were not able to detect 1XE2C driven by the *ade6* promoter by microscopy, but could detect it easily when driven by the *act1* promoter (high expression) and when fused C-terminally to Swi6 (moderate expression). SF-GFP and E2C are clearly most ideal for experiments aimed at dynamics. For steady state reporter gene approaches, all three XFPs are useful and all can be detected simultaneously. We find that three-color detection can be achieved either with two lasers, where there is some cross bleed particularly from mKO2 into the E2C detection channels (Fig 2B and S1 Fig), or with three lasers, eliminating any significant cross bleed (Fig 2D). SF-GFP and mKO2 are most suitable for steady state microscopic imaging, especially of low abundance proteins. Plasmids allowing straightforward C-terminal XFP tagging of proteins of choice contain kan^R^, nat^R^ and hyg^R^ cassettes have been made available through Addgene.

Maturation times between XFPs varies widely, even within the same family [10, 14]. The question of XFP maturation times in the system of study is largely unaddressed in most studies, yet knowing the delay between protein production and fluorescence is necessary to interpret time-dependent data. It has widely been assumed the maturation times can be extracted from *in vitro* measurements, or that maturation parameter derived from one system (typically *E*.*coli*) would apply to the system of study. However, given recent findings that there are differences maturation times even between strains of the same organism [20], it is reasonable to consider that maturation times may vary between organisms. This is on one level not surprising, as chromophore formation is pH sensitive [22–24] and widely known to be temperature sensitive [21]. However, at least in the case of GFP, no dependence on other cofactors has been demonstrated [42]. But these two factors alone, pH and growth temperature, can vary considerably between model organisms. Thus, it appears a common method for determining the *in vivo* maturation rate might be useful to interpret XFP kinetic data. Such a common framework with some variations exists for *E*.*coli*, where XFP expression is induced by IPTG and translation is shut off with chloramphenicol [12]. In this pulse-chase scenario, folding and maturation of a defined protein quantity is followed. However, if the protein accumulation is not measured, inducer response and translation will be confounded with folding and maturation. When fluorescence can be measured at high sensitivity, it is also possible to measure maturation times by following the increase over steady state following the addition of a translation block. Yet, this is not always a practical approach as appropriately sensitive equipment is not widely available.

We utilized a simple approach to measure maturation times *in vivo* in *S*.*pombe*. The approach has the following parts (Fig 2A): Induction of a XFP:5XFLAG message in response to a soluble inducer (uracil) for a short time window (pulse of protein production), measurement of protein and fluorescence accumulation, and finally fitting of folding and maturation kinetics. While conceptually any inducible system should do, a unique and useful feature of the *urg1* gene system we use is that the message is very unstable following uracil removal (Fig 3 and [35]), guaranteeing a defined burst of message production. By simultaneously integrating total protein information into the fluorescence fit (see methods), we are able to account for any delays in induction and consequentially, protein production, which might arise from experiment to experiment or strain to strain variation. The importance of this approach is validated by the slow protein induction of SF-GFP as opposed to E2C or mKO2 (Figs 3C and 4A bottom). Importantly, using a mathematical approach we were able to show that the slow appearance of mKO2 fluorescence following induction is the result of the slow production of a non-fluorescent intermediate as well as a relatively slow maturation rate. We expanded the model to a three protein state model for mKO2 and we believe such a model delivers meaningful *in vivo* of *k*_*F*_ and *k*_*M*_ values given the highly constrained parameter space these variables can adopt resulting in robust data fits (S3 Fig). Given that the rates obtained here are derived in the context of arbitrary fluorescence units, they cannot be directly compared to data obtained under experimental conditions divergent from those described here. However, they are powerful in allowing quantitative comparison between fluors using internally consistent experimental approach.

The experimental approach should be easy to replicate for other XFP proteins in *S*.*pombe*, and for other biological systems, such as *Saccharomyces cerevisiae* that for which appropriate induction systems are available.

## Materials and Methods

### Strain source, construction and growth

All parent *S*. *pombe* strains used for this study are derivatives of publically available strains. The genotypes of all strains used in this study can be found in Table 1. The *ade6p*:XFP:*ura4t*:hygMX and *act1p*:XFP:*ura4t*:hygMX cassettes were produced using *in vivo* recombination techniques and introduced in between SPBC1711.11 and SPBC1711.12 in the BAS99 background. To produce *urg1p*:XFP:5xFLAG strains, we first disrupted the *Urg1* open reading frame with a KanMX cassette (Δ*urg1*::KanMX). We replaced the KanMX cassette with a *urg1p*:XFP:5xFLAG construct that carried a NatMX cassette. To minimize interfere of the MX cassette with the *urg1* locus, we included in the construct 1.6kb of *urg1* 3’ UTR downstream of the XFP insertion site before the beginning of the MX cassette. To produce mKO_s.p._:Dcr1 fusion proteins driven from the endogenous *dcr1* promoter, we inserted a KanMX marked construct upstream of *Dcr1* that contained mKO2_s.p._ inserted between the endogenous *dcr1* promoter and the *Dcr1* ORF. To produce *swi6*::*swi6*:1XE2C, *swi6*::*swi6*:SF-GFP and *sad1*::*sad1*:mKO2, the appropriate cassette in Fig 1 was PCR amplified with 80bp homology primers and inserted into the endogenous locus by homologous recombination. The double labeled *swi6*::*swi6*:SF-GFP; *sad1*::*sad1*:mKO2 strain was produced by a genetic cross. For all experiments, *S*.*pombe* cells were grown in EMM media at 32°C.

### Flow cytometry

All strains were grown in EMM media overnight to about ~OD 1.5 and were back diluted to OD 0.1 and grown for an additional 3-5hrs before flow cytometry. All analysis was performed in the UCSF Parnassus Flow Cytometry core on Beckton Dickinson Flow LSRII or Fortessa cytometers outfitted with 488nm, 532 or 561nm, and 640nm lasers. PMT voltages were adjusted as to yield a fluorescence value of 10^2^ for untransformed cells in each channel. Cells were gated for single recently divided cells by their forward and side scatter characteristics and their mean fluorescence values reported.

### Uracil induction

Cells were grown through the day in EMM-uracil to about OD 0.5 and diluted to 0.01 for overnight growth. The next morning, cells were typically in early log phase and induced for 20min with 0.25mg/ml uracil. Uracil was removed by centrifugation of the induced cultures, one wash in PBS, and reintroduction of EMM-uracil media.

### Western blots

For uracil induction time-courses, cells were flash-frozen and total protein extracted using previously described methods [43]. Low-fluorescence PVDF membranes (Bio-rad) were probed with polyclonal anti-FLAG (Abgent AP1013a) and monoclonal anti GAPDH (Thermo Scientific MA5-15738) primary antisera simultaneously, detected by IR-dye linked 680 and 800nm secondary antibodies and antibody fluorescence analyzed as described [44]. FLAG signals were normalized to GAPDH loading control signals.

### Real-time qPCR

Total RNA was extracted from yeast cells using the Masterpure yeast total RNA kit (Illumina). 3μg RNA was reverse transcribed with Superscript III reverse transcriptase (Invitrogen). RT-qPCR was performed as described using an actin internal control [44, 45]

### Fitting

For SF-GFP and E2C, we derived the following overall equations for a two state consecutive reaction model following uracil induction:

XFP RNA (*RNA*) ➔ XFP total protein (*P*_*T*_) ➔ XFP folded/matured (*P*_*FM*_):

In this two protein state model, *P*_*T*_ *= P*_*U*_ *+ P*_*FM*_, where *P*_*T*_ is total protein, *P*_*U*_ is unfolded, non-fluorescent protein, *P*_*FM*_ is folded and mature fluroescent protein. We directly measure *P*_*T*_ and *P*_*FM*_. The equations describing these sequential reactions are described by the following Ordinary Differential Equations (ODEs):

$$(1)\;\frac{dRNA}{dt}=u-\alpha_{m}RNA$$

$$(2)\;\frac{dP_{T}}{dt}=k_{P}RNA-\alpha_{p}P_{T}$$

$$(3)\;\frac{dP_{M}}{dt}=k_{FM}(\theta P_{T}-P_{M})-\beta_{FM}P_{M}$$

In this model, *u* represents the rate constant of mRNA induction by uracil, and *α*_*m*_ the rate constant of RNA turnover. *P*_*T*_ denotes total protein as measured by western blotting. *k*_*P*_ and *α*_*p*_ are the rate constants of translation and total protein degradation, respectively. *P*_*M*_ denotes matured protein as measured by fluorescence. *k*_*FM*_ and *β*_*FM*_ are the rate constants of folding/maturation and fluorescent protein degradation, respectively. *θ* is the scaling factor between protein units (FLAG/GAPDH) and fluorescence units (arbitrary fluorescence units).

We fit the total protein time course data to equation *(2)* and the fluorescence time course data to *(3)*. In the fitting for *(3)*, we use the *P*_*T*_ values from the fit of the total protein data to *(2)* as inputs.

For SF-GFP, given the delayed kinetics of protein appearance (see results) we introduced a step depicting transitions between closed to open chromatin preceding transcription. In this model, chromatin transition to the open state (forward arrow) is influenced by the uracil input (*u*), while transition to the closed state (back arrow) is governed by *k*_*c*_.

$$C_{c}⇌C_{o}$$

*C*_*c*_ is the closed, non-induction competent state, and *C*_*o*_ is the open, induction competent state. To model this step, we introduce an additional equation for the dynamics of the open chromatin state:

$$\frac{{dC}_{o}}{dt}=u(1-C_{o})-k_{c}C_{o}$$

Also using that *C*_*o*_ + *C*_*c*_ = 1. Since transcription is only possible from *C*_*o*_, We modified equation *(1)* above for SF-GFP:

$$\frac{dRNA}{dt}=k_{o}C_{o}-\alpha_{m}RNA$$

Where *k*_*o*_ is the rate of transcription from the open chromatin state.

For mKO2, since a model aggregating maturation and folding of the fluorescent protein could not reproduce the data, we expanded the model to include separate steps for these processing, resulting in a three protein sequential reaction model following uracil induction:

mKO2 RNA (*RNA*) ➔ total protein (*P*_*T*_) ➔ folded protein (*P*_*F*_) ➔ mKO2 mature protein (*P*_*M*_)

In this protein state model, *P*_*T*_ *= P*_*U*_ *+ P*_*F*_ *+ P*_*M*_, where *P*_*T*_ is total protein, *P*_*U*_ is unfolded, non-fluorescent protein, *P*_*F*_ is folded, non-fluorescent protein and *P*_*M*_ is mature, fluroescent protein.

$$(1)\;\frac{dRNA}{dt}=u-\alpha_{m}RNA$$

$$(2)\;\frac{dP_{T}}{dt}=k_{P}RNA-\alpha_{p}P_{T}$$

$$(3)\;\frac{dP_{F}}{dt}=k_{F}(\theta P_{T}-P_{F}-P_{M})-k_{M}P_{F}{-\beta }_{F}P_{F}$$

$$(4)\;\frac{dP_{F}}{dt}=k_{M}P_{F}{-\beta }_{M}P_{M}$$

The fitting was performed using a customized script within the MatLab (Mathworks) software. Briefly, we sought to find a large set of parameters that can fit the data within a pre-defined error tolerance. In this case, we defined the error tolerance as the mean of the absolute value of all the errors between the fit and individual data points. We set this tolerance to 5%. To generate the model parameters that can fit the data, we fed different initial guesses to the function fminsearch, which uses a nonlinear Nelder-Mead algorithm to find minima of a function. We then used the successful guesses as “seeds” for another algorithm that expands around the successful parameters sets, trying different combinations and storing the ones that satisfy the error tolerance.

### Microscopy

*S*.*pombe* cells were grown as above for flow cytometry measurements. Glass slides were coated with concanavalin A, were cells applied to the slide and imaged using a Yokagawa CSU22 Spinning Disc confocal fitted Nikon Ti-E microscope. In the “DAPI” channel (for DAPI), excitation was performed by a 405nm laser and the emission preset was 460/50nm. In the “GFP” channel (for SF-GFP) excitation was performed by a 488nm laser and the emission preset was 525/50nm. In the “RFP” channel (for mKO2 and E2C) excitation was performed by a 561nm laser and the emission preset was 610/60nm. In the “Cy5” channel (for E2C) excitation was performed by a 640nm laser and the emission preset was 700/75nm.

### Image processing

Images were false colored in ImageJ software and merged. The brightness adjusted for the separate channels so no background color (intensity outside the cell) dominates in the merged image. Channels were then split and images saved as jpegs. Contrast was adjusted for clarity across the entire image. For mKO2:Dcr1 cell images, the RFP channel was smoothened given the low signal.

## Supporting Information

S1 Fig Compensation for cross-bleed using 488nm/561nm/561nm excitation regime. Shown on the left column are uncompensated, raw histograms of unstained, SF-GFP_s.p._, mKO2_s.p._ and 3xE2C_s.p._ cells with the emissions filter indicated on the bottom excited with either the 561nm (TOP, BOTTOM) or 488nm (MIDDLE) laser. Significant bleed from mKO2_s.p._ (orange arrows)into the E3C channel and slight bleed into the SF-GFP channel is evident. The right column shows the corresponding histograms following compensation by the Becton Dickinson FACSDiva software. All cross bleed by mKO2_s.p._ is eliminated.
(PDF)

S2 Fig Compensation for cross-bleed using 488nm/561nm/640nm excitation regime. As in S1 Fig, expect that excitation was performed by three lasers (488 for SF-GFP, 561 for mKO2 and 640 for 3XE2C). Only slight bleed from mKO2_s.p._ into the SF-GFP channel is evident and eliminated upon compensation.
(PDF)

S3 Fig Parameter distributions for fits in Fig 4. **A.** Parameters distributions for E2C_s.p._ total protein (LEFT) and fluorescence (RIGHT) fits that yield a fit with <5% mean fitting error. **B.** Parameters distributions for SF-GFP_s.p._ total protein including open and closed chromatin transitions, (LEFT) and fluorescence (RIGHT) fits that yield a fit with <5% mean fitting error. **A.** Parameters distributions for mKO2_s.p._ total protein (LEFT) and fluorescence (RIGHT) fits that yield a fit with <5% mean fitting error. All *α*, *β* and *k* values are in min^-1^.
(PDF)

S4 Fig Second run of mKO2 induction and alternative fitting approach. **A.** Shown is a second repeat of uracil pulse induction of *urg1p*::mKO2_s.p._ Expression was induced, protein and fluorescence analyzed and fit as in Fig 4B using the three protein state model. The mean fit is shown in color, the gray lines are all possible fits. **B.** Fitting mKO2_s.p._ fluorescence with a 2 protein state model with a delay function. We derived a single *k*_F/M*_ form the same raw data as shown in Fig 3 and Fig 4B using a two protein state model and delay.
(PDF)

S5 Fig Single channel microscopy with SF-GFP, mKO2 and 3XE2C and visualization of Dcr1 with mKO2. **A.** Untagged *ade6* promoter driven XFPs. **TOP:** SF-GFP_s.p._ cells were visualized in brightfield, the GFP, RFP and Cy5 channels. **Middle:** mKO2_s.p._ cells were visualized in brightfield, the GFP, RFP and Cy5 channels. Images were taken at 60x magnification. **Bottom:** 3XE2C_s.p._ cells were visualized in brightfield, the GFP, RFP and Cy5 channels. **B.** C-terminally Visualization of mKO2:Dcr1 driven by its endogenous promoter. mKO2 was inserted between the *dcr1* promoter and the *dcr1* open reading frame and tagged upstream with a G418 resistance cassette. Cells were stained with DAPI and visualized in the DAPI and RFP channel. Two fields of cells are shown. Arrowheads denote location of cell nuclei. Image were taken at 100x magnification.
(PDF)

S1 File Alignments of *S*.*pombe*. (*s*.*p*.) codon optimized to non-optimized sequences. (DOCX)
