# Supplementary figures and images for: Sensitive and Quantitative Three-Color Protein Imaging in Fission Yeast Using Spectrally Diverse, Recoded Fluorescent Proteins with Experimentally-Characterized In Vivo Maturation Kinetics
Source: PLoS One. 2016 Aug 1;11(8):e0159292. doi: 10.1371/journal.pone.0159292 (PMC4968791; doi:10.1371/journal.pone.0159292)

# SFigure 1

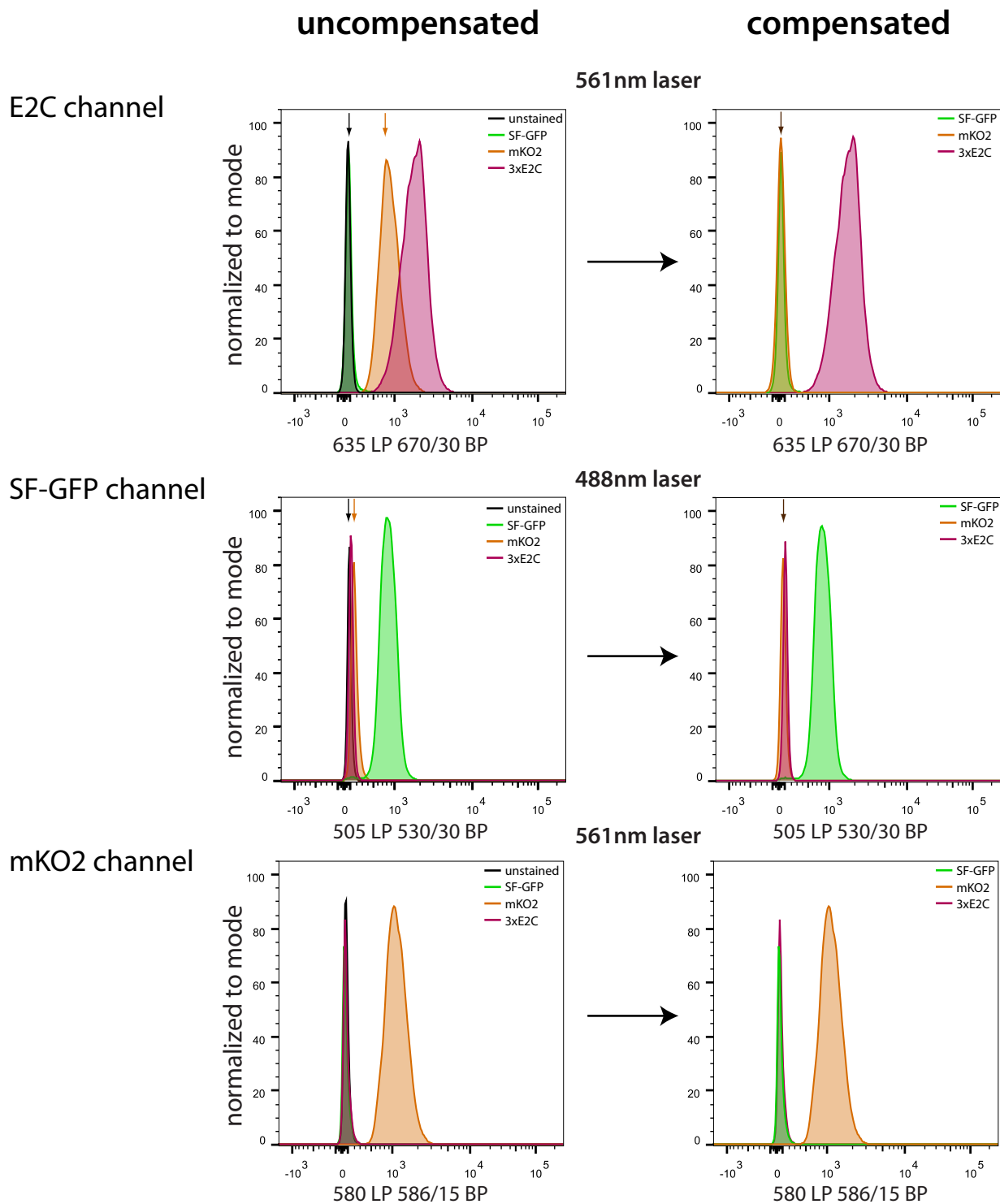

Supplement: S1 Fig — Shown on the left column are uncompensated, raw histograms of unstained, SF-GFPs.p., mKO2s.p. and 3xE2Cs.p. cells with the emissions filter indicated on the bottom excited with either the 561nm (TOP, BOTTOM) or 488nm (MIDDLE) laser. Significant bleed from mKO2s.p. (orange arrows)into the E3C channel and slight bleed into the SF-GFP channel is evident. The right column shows the corresponding histograms following compensation by the Becton Dickinson FACSDiva software. All cross bleed by mKO2s.p. is eliminated. (PDF) [file pone.0159292.s001.pdf]

SFigure 2

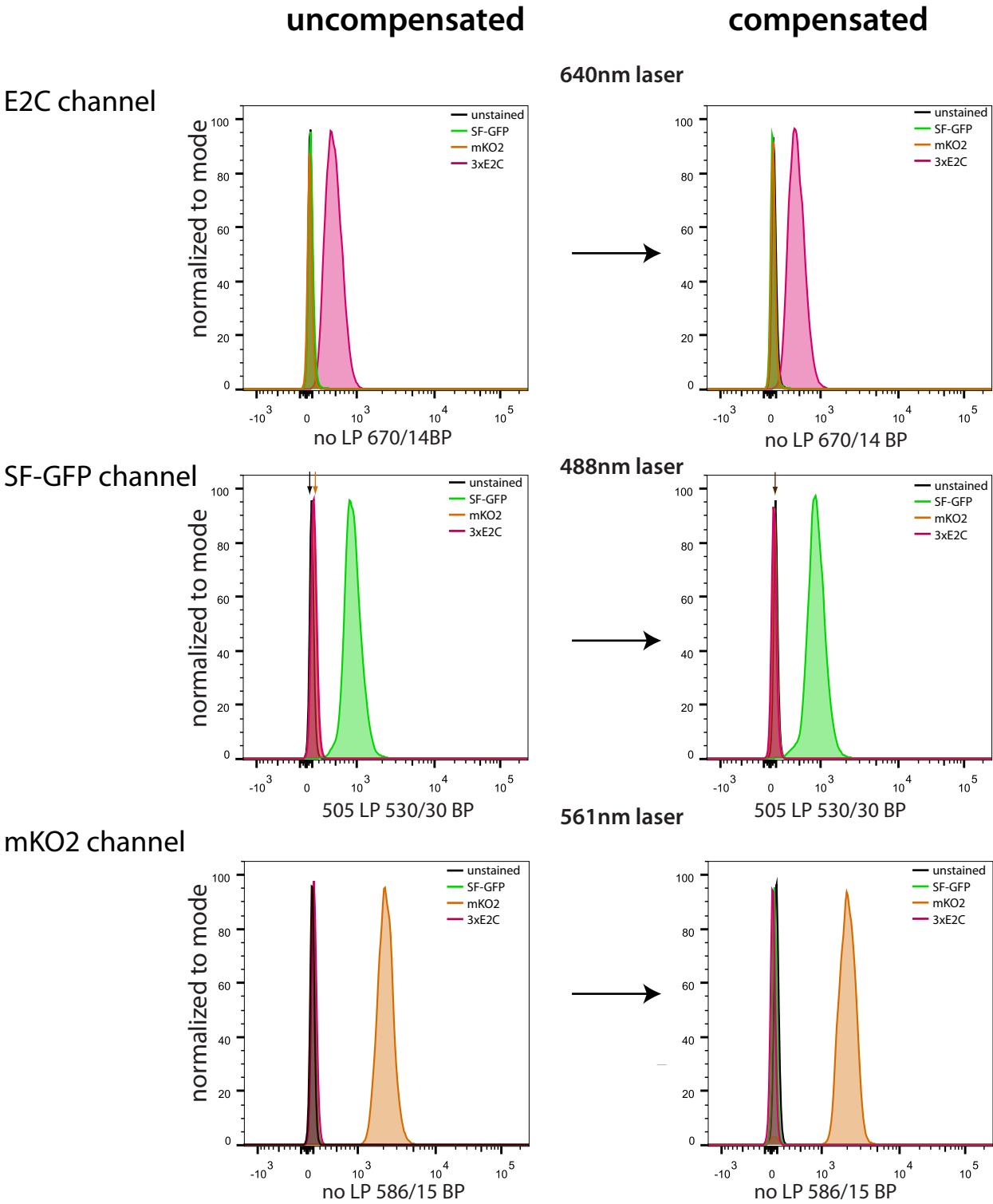

Supplement: S2 Fig — As in S1 Fig, expect that excitation was performed by three lasers (488 for SF-GFP, 561 for mKO2 and 640 for 3XE2C). Only slight bleed from mKO2s.p. into the SF-GFP channel is evident and eliminated upon compensation. (PDF) [file pone.0159292.s002.pdf]

# SFigure 4

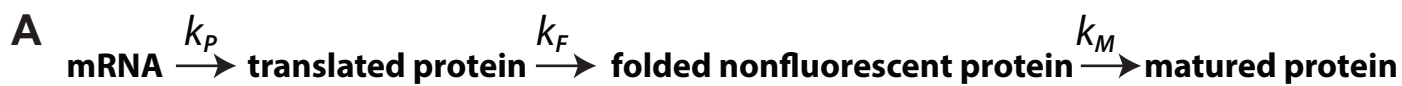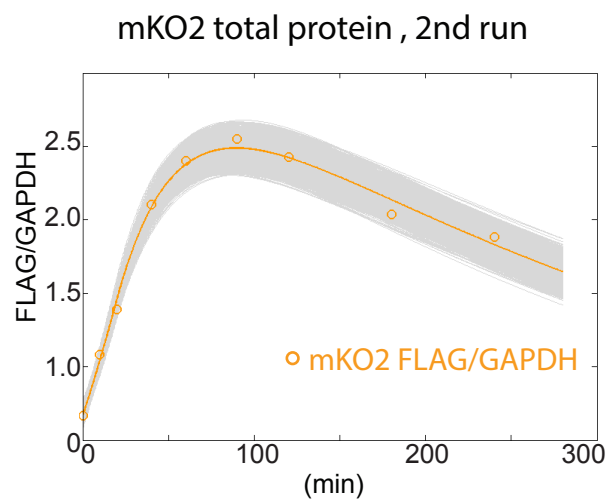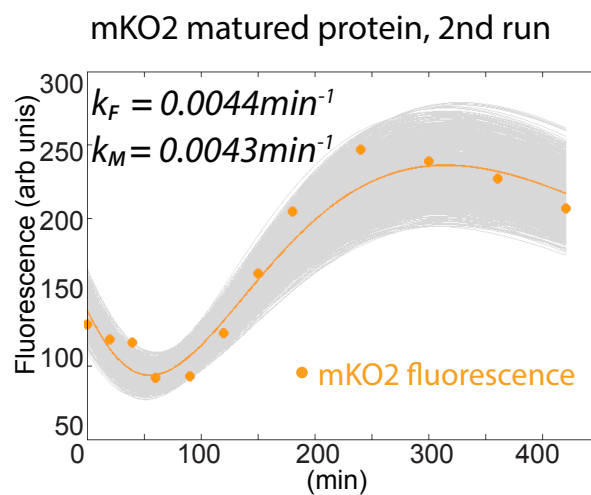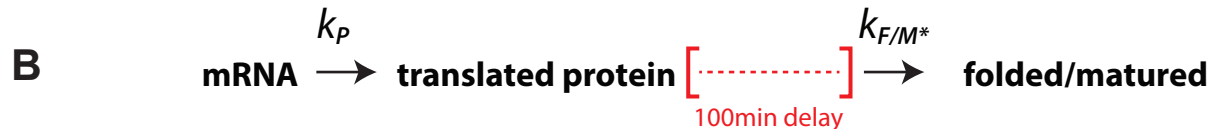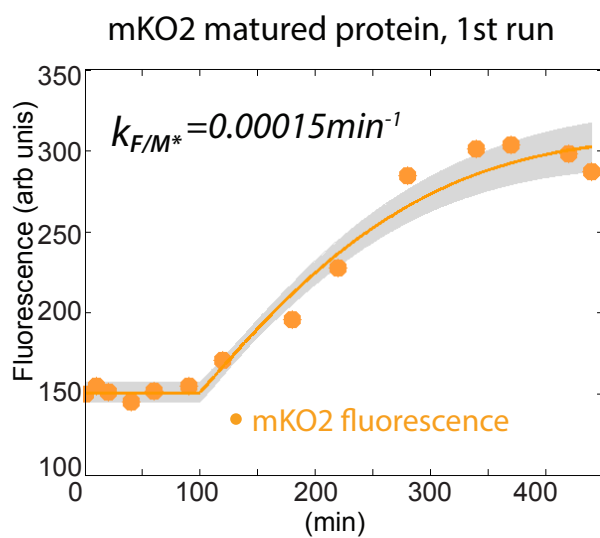

Supplement: S4 Fig — A. Shown is a second repeat of uracil pulse induction of urg1p::mKO2s.p. Expression was induced, protein and fluorescence analyzed and fit as in Fig 4B using the three protein state model. The mean fit is shown in color, the gray lines are all possible fits. B. Fitting mKO2s.p. fluorescence with a 2 protein state model with a delay function. We derived a single kF/M* form the same raw data as shown in Fig 3 and Fig 4B using a two protein state model and delay. (PDF) [file pone.0159292.s004.pdf]

# Supplementary Figure 5

A

ade6p:SF-GFP

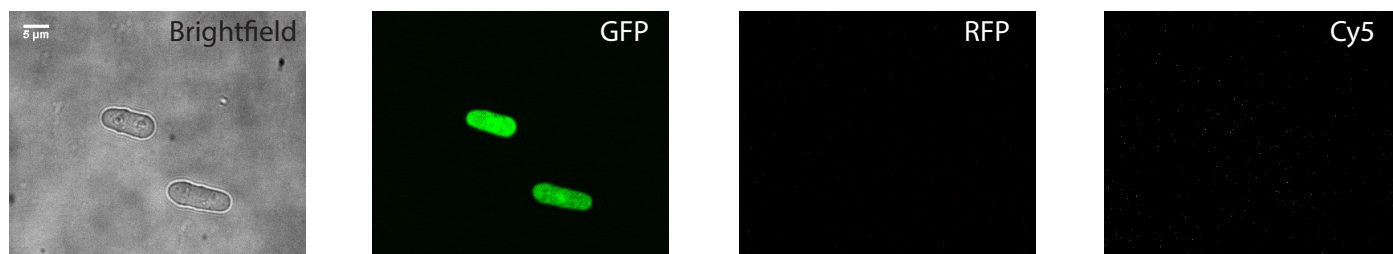

ade6p:mKO2

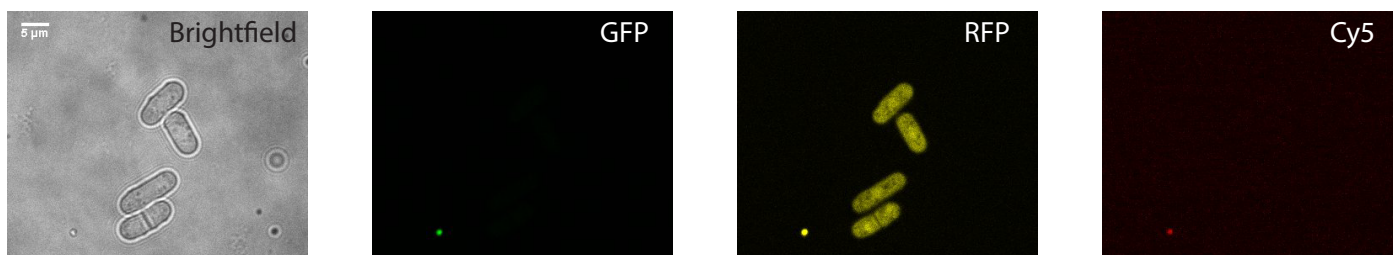

ade6p:3XE2C

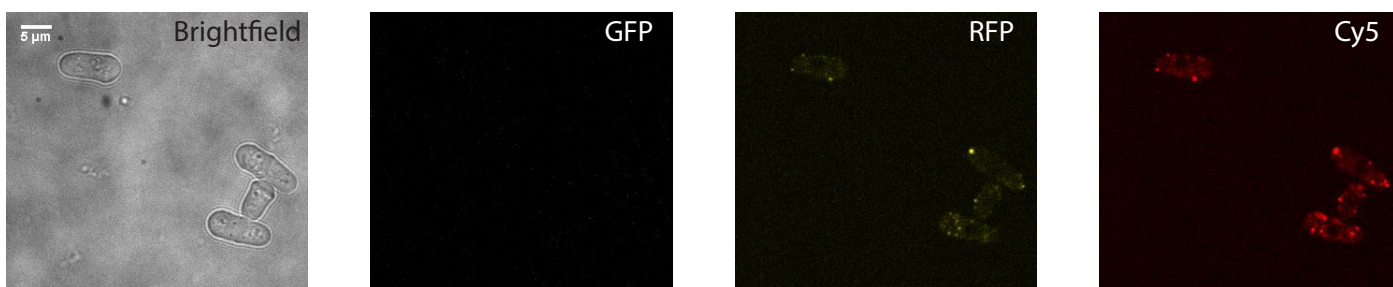

B

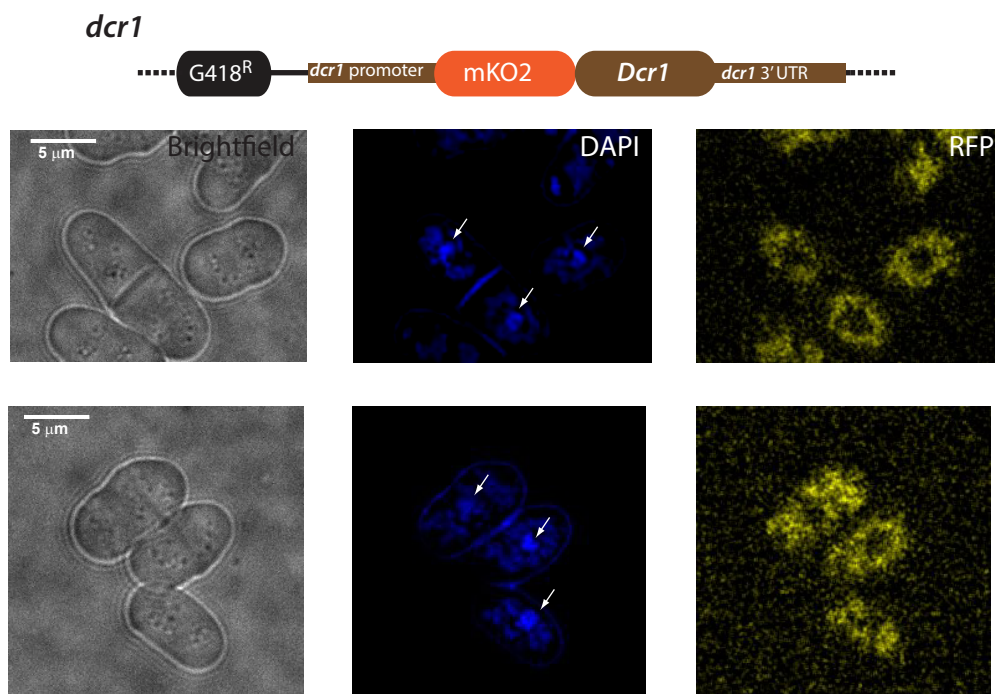

Supplement: S5 Fig — A. Untagged ade6 promoter driven XFPs. TOP: SF-GFPs.p. cells were visualized in brightfield, the GFP, RFP and Cy5 channels. Middle: mKO2s.p. cells were visualized in brightfield, the GFP, RFP and Cy5 channels. Images were taken at 60x magnification. Bottom: 3XE2Cs.p. cells were visualized in brightfield, the GFP, RFP and Cy5 channels. B. C-terminally Visualization of mKO2:Dcr1 driven by its endogenous promoter. mKO2 was inserted between the dcr1 promoter and the dcr1 open reading frame and tagged upstream with a G418 resistance cassette. Cells were stained with DAPI and visualized in the DAPI and RFP channel. Two fields of cells are shown. Arrowheads denote location of cell nuclei. Image were taken at 100x magnification. (PDF) [file pone.0159292.s005.pdf]
