# Supplementary material for: Sensitive and Quantitative Three-Color Protein Imaging in Fission Yeast Using Spectrally Diverse, Recoded Fluorescent Proteins with Experimentally-Characterized In Vivo Maturation Kinetics
Source: PLoS One. 2016 Aug 1;11(8):e0159292. doi: 10.1371/journal.pone.0159292 (PMC4968791; doi:10.1371/journal.pone.0159292)
Supplement: S3 Fig — A. Parameters distributions for E2Cs.p. total protein (LEFT) and fluorescence (RIGHT) fits that yield a fit with <5% mean fitting error. B. Parameters distributions for SF-GFPs.p. total protein including open and closed chromatin transitions, (LEFT) and fluorescence (RIGHT) fits that yield a fit with <5% mean fitting error. A. Parameters distributions for mKO2s.p. total protein (LEFT) and fluorescence (RIGHT) fits that yield a fit with <5% mean fitting error. All α, β and k values are in min-1. (PDF) [file pone.0159292.s003.pdf]

SFigure 3

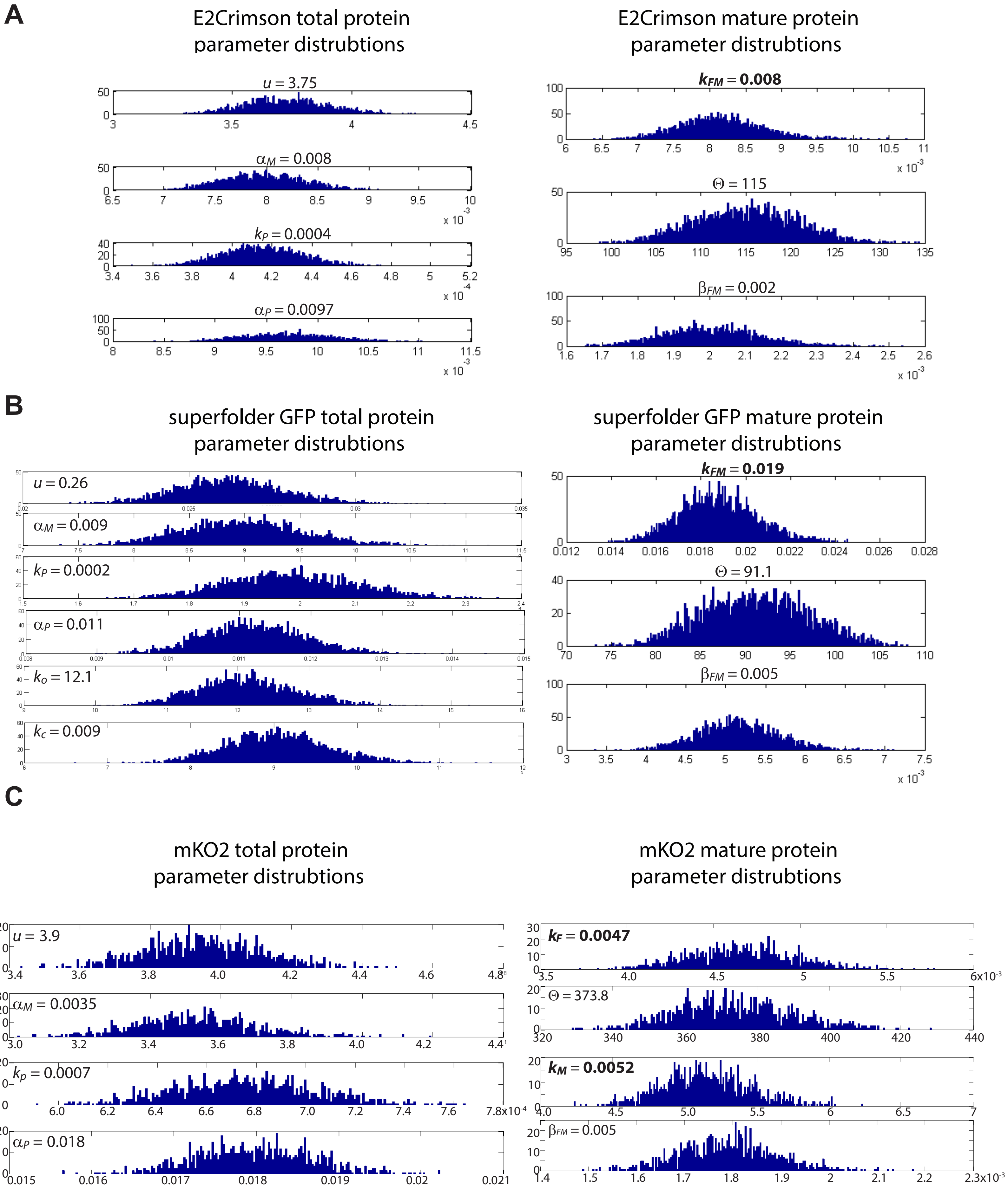

A
E2Crimson total protein parameter distrubtions
u = 3.75
αM = 0.008
kP = 0.0004
αP = 0.0097
E2Crimson mature protein parameter distrubtions
kFM = 0.008
Θ = 115
βFM = 0.002
B
superfolder GFP total protein parameter distrubtions
u = 0.26
αM = 0.009
kP = 0.0002
αP = 0.011
ko = 12.1
kc = 0.009
superfolder GFP mature protein parameter distrubtions
kFM = 0.019
Θ = 91.1
βFM = 0.005
C
mKO2 total protein parameter distrubtions
u = 3.9
αM = 0.0035
kp = 0.0007
αP = 0.018
mKO2 mature protein parameter distrubtions
kF = 0.0047
Θ = 373.8
kM = 0.0052
βFM = 0.005
