# Supplementary material for: Sensitive and Quantitative Three-Color Protein Imaging in Fission Yeast Using Spectrally Diverse, Recoded Fluorescent Proteins with Experimentally-Characterized In Vivo Maturation Kinetics
Source: PLoS One. 2016 Aug 1;11(8):e0159292. doi: 10.1371/journal.pone.0159292 (PMC4968791; doi:10.1371/journal.pone.0159292)
Supplement: S1 File — (DOCX) [file pone.0159292.s006.docx]

**S1 File:**

**Alignments of *S.pombe.* (*s.p.*) codon optimized to non-optimized sequences (|, identity, . change)**

Bassem Al-Sady^1*^, Rachel A Greenstein^1,3^ , Hana J El-Samad^2^, Sigurd Braun^4^ and Hiten D Madhani^2*^

mKO2 1 atggtttctgtgatcaagcccgaaatgaagatgaggtactacatggacgg 50

|||||||||||.||.|||||.||||||||||||.|.|||||.|||||.||

mKO2s.p. 1 ATGGTTTCTGTTATTAAGCCTGAAATGAAGATGCGTTACTATATGGATGG 50

mKO2 51 cagcgtcaatggacatgagtttacgatcgaaggggagggcaccggccgac 100

....||.|||||.|||||.|||||.||.|||||.||.||.||.||.||.|

mKO2s.p. 51 TTCTGTTAATGGTCATGAATTTACTATTGAAGGTGAAGGTACTGGTCGTC 100

mKO2 101 cctacgagggccaccaggagatgaccctgagggtgacgatggcagagggc 150

|.||.||.||.||.||.||.|||||.||..|.||.||.|||||.||.||.

mKO2s.p. 101 CTTATGAAGGTCATCAAGAAATGACTCTTCGTGTTACTATGGCTGAAGGT 150

mKO2 151 ggtcccatgcccttcgccttcgacctggtaagccacgtgttctgctacgg 200

|||||.|||||.||.||.||.||.||.||....||.||.||.|||||.||

mKO2s.p. 151 GGTCCTATGCCTTTTGCTTTTGATCTTGTTTCTCATGTTTTTTGCTATGG 200

mKO2 201 ccaccgagtgttcacaaaatacccggaggagatcccagactacttcaagc 250

.||.||.||.||.||.||.||.||.||.||.||.||.||.|||||.||||

mKO2s.p. 201 TCATCGTGTTTTTACTAAGTATCCTGAAGAAATTCCTGATTACTTTAAGC 250

mKO2 251 aggcctttcccgaggggctgagctgggagaggagcctggaatttgaggac 300

|.||.|||||.||.||.||....|||||..|....||.||||||||.||.

mKO2s.p. 251 AAGCTTTTCCTGAAGGTCTTTCTTGGGAACGTTCTCTTGAATTTGAAGAT 300

mKO2 301 ggtggcagtgctagcgttagcgcccacatcagtctgaggggcaacacctt 350

|||||...||||...|||...||.||.||...|||..|.||.||.||.||

mKO2s.p. 301 GGTGGTTCTGCTTCTGTTTCTGCTCATATTTCTCTTCGTGGTAATACTTT 350

mKO2 351 ttaccacaagagcaagttcactggcgttaacttcccagcggacggcccca 400

||||||.|||...|||||.|||||.|||||.||.||.||.||.||.||.|

mKO2s.p. 351 TTACCATAAGTCTAAGTTTACTGGTGTTAATTTTCCTGCTGATGGTCCTA 400

mKO2 401 tcatgcagaaccagagcgtggactgggagcccagcaccgagaagatcacc 450

|.|||||.||.||....||.||.|||||.||....||.||.|||||.||.

mKO2s.p. 401 TTATGCAAAATCAATCTGTTGATTGGGAACCTTCTACTGAAAAGATTACT 450

mKO2 451 gccagcgacggcgtgctgaagggcgacgtgaccatgtacctgaagctgga 500

||....||.||.||.||.|||||.||.||.||.||||||||.|||.|.||

mKO2s.p. 451 GCTTCTGATGGTGTTCTTAAGGGTGATGTTACAATGTACCTTAAGTTAGA 500

mKO2 501 aggaggtggcaaccataagtgccagatgaagaccacctacaaggccgcca 550

|||.|||||.||||||||||||||.||||||||.||.||||||||.||.|

mKO2s.p. 501 AGGTGGTGGTAACCATAAGTGCCAAATGAAGACTACTTACAAGGCTGCTA 550

mKO2 551 aggagattctggagatgccgggagaccactatattggacacaggctggtc 600

|.||.|||||.||.|||||.||.||.||.||.||||||||..|.||.||.

mKO2s.p. 551 AAGAAATTCTTGAAATGCCTGGTGATCATTACATTGGACATCGTCTTGTT 600

mKO2 601 cgaaagactgaaggtaacatcaccgaacaggtggaagacgccgtggccca 650

||.|||||||||||||||||.||.|||||.||.|||||.||.||.||.||

mKO2s.p. 601 CGTAAGACTGAAGGTAACATTACTGAACAAGTTGAAGATGCTGTTGCTCA 650

mKO2 651 ctacagctaa 660

.||....|||

mKO2s.p. 651 TTATTCTTAA 660

E2C 1 atggattcaactgagaacgtgatcaagccattcatgaggttcaaggtgca 50

||||||||.|||||.|||||.||.|||||.||.|||.|.||.|||||.||

E2Cs.p. 1 ATGGATTCTACTGAAAACGTTATTAAGCCTTTTATGCGTTTTAAGGTTCA 50

E2C 51 catggagggcagcgtgaacggacacgagttcgaaatagagggcgtgggcg 100

.|||||.||....||.||.||.||.||.||.|||||.||.||.||.||.|

E2Cs.p. 51 TATGGAAGGTTCTGTTAATGGTCATGAATTTGAAATTGAAGGTGTTGGTG 100

E2C 101 aaggcaagccctacgagggcacccagaccgccaagctgcaggtgaccaag 150

||||.|||||.||.||.||.||.||.||.||.|||||.||.||.||.||.

E2Cs.p. 101 AAGGTAAGCCTTATGAAGGTACTCAAACTGCTAAGCTTCAAGTTACAAAA 150

E2C 151 gggggaccactgccgttcgcttgggacatcctgagcccccagttcttcta 200

||.||.||..|.||.||.||||||||.||.||....||.||.||.||.||

E2Cs.p. 151 GGTGGTCCTTTACCTTTTGCTTGGGATATTCTTTCTCCTCAATTTTTTTA 200

E2C 201 cggcagcaaggcctacattaagcatcccgccgacatccccgactatctga 250

|||....||.||.||||||||||||||.||.||.||.||.||.||.||.|

E2Cs.p. 201 CGGTTCTAAAGCTTACATTAAGCATCCTGCTGATATTCCAGATTACCTTA 250

E2C 251 agcagagcttccccgagggcttcaagtgggagagggtgatgaatttcgag 300

||||....||.||.||.||.||.||||||||..|.||.|||||.||.||.

E2Cs.p. 251 AGCAATCATTTCCTGAAGGTTTTAAGTGGGAACGTGTTATGAACTTTGAA 300

E2C 301 gatggcggcgtcgtaaccgtgacccagga--cagcagcctgcaagacggc 348

|||||.||.||.||.||.||.||.||.|| |..|| ||.|||||.||.

E2Cs.p. 301 GATGGTGGTGTTGTTACTGTTACTCAAGATTCTTCA--CTTCAAGATGGT 348

E2C 349 accctgatctaccacgtgaagttcatcggcgtaaacttccccagcgatgg 398

||.||.||.|||||.||.|||||.||.||.||.||.||.||....|||||

E2Cs.p. 349 ACTCTTATTTACCATGTTAAGTTTATTGGTGTTAATTTTCCTTCTGATGG 398

E2C 399 ccccgtgatgcagaagaagaccctgggctgggagcccagcaccgagagga 448

.||.||.|||||.||.|||||..|.||.|||||.||....||.||..|.|

E2Cs.p. 399 TCCTGTTATGCAAAAAAAGACTTTAGGTTGGGAACCTTCTACAGAACGTA 448

E2C 449 actaccccagggacggcgtgctgaaaggcgagaaccacatggcactgaag 498

|.||.||..|.||.||.||.||.||.||.||.||.||.|||||.||.|||

E2Cs.p. 449 ATTATCCTCGTGATGGTGTTCTTAAGGGTGAAAATCATATGGCTCTTAAG 498

E2C 499 ctgaagggagggggccactacctgtgcgagttcaagagcatctacatggc 548

.||||.||.||.||.||.|||||.|||||.||.|||...||.||||||||

E2Cs.p. 499 TTGAAAGGTGGTGGTCATTACCTTTGCGAATTTAAGTCTATTTACATGGC 548

E2C 549 caagaagcccgtgaaactgcccggctaccactacgtggactacaagctgg 598

.||||||||.||.||.||.||.||.||.||.|||||.||.||||||||.|

E2Cs.p. 549 TAAGAAGCCTGTTAAGCTTCCTGGTTATCATTACGTTGATTACAAGCTTG 598

E2C 599 acatcaccagccacaacgaggactatacagttgtggagcagtacgaacgc 648

|.||.||....||.||.||.||.|||||.|||||.||.||.||.|||||.

E2Cs.p. 599 ATATTACTTCTCATAATGAAGATTATACTGTTGTTGAACAATATGAACGT 648

E2C 649 gctgaggccaggcaccacctgttccagtaa 678

|||||.||..|.||.||.||.||.||.|||

E2Cs.p. 649 GCTGAAGCTCGTCATCATCTTTTTCAATAA 678

SF-GFP 1 ATGGTGTCCAAGGGCGAGGAGCTGTTCACCGGCGTGGTGCCCATCCTGGT 50

|||||.||.|||||.||.||.||.||.||.||.||.||.||.||.||.||

SF-GFPs.p. 1 ATGGTTTCTAAGGGTGAAGAACTTTTTACTGGTGTTGTTCCTATTCTTGT 50

SF-GFP 51 GGAGCTGGATGGCGACGTGAACGGCCACAAGTTCAGCGTGCGCGGCGAGG 100

.||.||.|||||.||.||.|||||.||.|||||....||.||.||.||.|

SF-GFPs.p. 51 TGAACTTGATGGTGATGTTAACGGTCATAAGTTTTCTGTTCGTGGTGAAG 100

SF-GFP 101 GCGAGGGCGACGCCACCAACGGCAAGCTGACCCTGAAGTTCATCTGCACC 150

|.||.||.||.||.||.||.||.||.||.||.||.|||||.||.|||||.

SF-GFPs.p. 101 GTGAAGGTGATGCTACTAATGGTAAACTTACTCTTAAGTTTATTTGCACT 150

SF-GFP 151 ACCGGCAAGCTGCCCGTGCCCTGGCCCACCCTGGTGACCACCCTGACCTA 200

||.||.|||.||||.||.||.|||||.||..|.||.||.||.||.||.||

SF-GFPs.p. 151 ACTGGTAAGTTGCCTGTTCCTTGGCCTACTTTAGTTACTACTCTTACTTA 200

SF-GFP 201 CGGCGTGCAGTGCTTCAGCCGCTACCCCGATCACATGAAGCAGCACGATT 250

.||.||.||.|||||....||.||.||.|||||.||||||||.||.||||

SF-GFPs.p. 201 TGGTGTTCAATGCTTTTCTCGTTATCCTGATCATATGAAGCAACATGATT 250

SF-GFP 251 TCTTCAAGAGCGCCATGCCCGAGGGCTACGTGCAGGAGCGCACCATCAGC 300

|.||.|||...||.|||||.||.||.||.||.||.||.||.||.||

SF-GFPs.p. 251 TTTTTAAGTCTGCTATGCCTGAAGGTTATGTTCAAGAACGTACTAT---- 296

SF-GFP 301 TTC----AAGGATGACGGCACCTACAAGACCCGCGCCGAGGTGAAGTTCG 346

||| ||||||||.||.||.||||||||.||.||.||.||.|||||.|

SF-GFPs.p. 297 TTCTTTTAAGGATGATGGTACTTACAAGACTCGTGCTGAAGTTAAGTTTG 346

SF-GFP 347 AGGGCGATACCCTGGTGAACCGCATCGAGCTGAAGGGCATCGATTTCAAG 396

|.||.|||||.||.||.||.||.||.||.||.|||||.||.|||||.||.

SF-GFPs.p. 347 AAGGTGATACTCTTGTTAATCGTATTGAACTTAAGGGTATTGATTTTAAA 396

SF-GFP 397 GAGGATGGCAACATCCTGGGCCACAAGCTGGAGTACAACTTCAACAGCCA 446

||.|||||.||.||.||.||.||.|||||.||.||||||||.||....||

SF-GFPs.p. 397 GAAGATGGTAATATTCTTGGTCATAAGCTTGAATACAACTTTAATTCTCA 446

SF-GFP 447 CAACGTGTACATCACCGCCGATAAGCAGAAGAACGGCATCAAGGCCAACT 496

.|||||.|||||.||.||.||||||||.||||||||.||.|||||.||||

SF-GFPs.p. 447 TAACGTTTACATTACTGCTGATAAGCAAAAGAACGGTATTAAGGCTAACT 496

SF-GFP 497 TCAAGATCCGCCACAATGTGGAGGATGGCTCCGTGCAGCTGGCCGATCAC 546

|.|||||.||.||.|||||.||.|||||.||.||.||.||.||.|||||.

SF-GFPs.p. 497 TTAAGATTCGTCATAATGTTGAAGATGGTTCTGTTCAACTTGCTGATCAT 546

SF-GFP 547 TACCAGCAGAACACCCCCATCGGCGACGGCCCAGTGCTGCTGCCCGATAA 596

|||||.||.|||||.||.||.||.||.||.||.||.||.||.||.|||||

SF-GFPs.p. 547 TACCAACAAAACACTCCTATTGGTGATGGTCCTGTTCTTCTTCCTGATAA 596

SF-GFP 597 CCACTACCTGAGCACCCAGAGC-GTGCTGTCCAAGGACCCCAACGAGAAG 645

|||.|||||....||.|| |.| ||.||.||.|||||.||.|||||.|||

SF-GFPs.p. 597 CCATTACCTTTCTACTCA-ATCTGTTCTTTCTAAGGATCCTAACGAAAAG 645

SF-GFP 646 CGCGATCACATGGTGCTGCTGGAGTTCGTGACCGCCGCCGGCATCACCCT 695

||.|||||.|||||.||..||||.||.||.||.||.||.||.||.||.||

SF-GFPs.p. 646 CGTGATCATATGGTTCTTTTGGAATTTGTTACTGCTGCTGGTATTACTCT 695

SF-GFP 696 GGGCATGGATGAGCTGTACAAGTAA 720

.||.||||||||.||.|||||||||

SF-GFPs.p. 696 TGGTATGGATGAACTTTACAAGTAA 720
